# Qin-Yu-Qing-Chang decoction reshapes colonic metabolism by activating PPAR-γ signaling to inhibit facultative anaerobes against DSS-induced colitis

**DOI:** 10.1186/s13020-024-01006-9

**Published:** 2024-09-26

**Authors:** Feng Xu, Jingyi Hu, Yanan Li, Cheng Cheng, Ryan Au, Yiheng Tong, Yuguang Wu, Yuan Cui, Yulai Fang, Hongxin Chen, Lei Zhu, Hong Shen

**Affiliations:** 1 Affiliated Hospital of Nanjing University of Chinese Medicine (Jiangsu Province Hospital of Chinese Medicine), Nanjing, 210029 China; 2 The First School of Clinical Medicine, Nanjing University of Chinese Medicine, Nanjing, 210023 China; 3 The Third Affiliated Hospital of Zhejiang Chinese Medical University, Hangzhou, 310005 China; 4 The Third Clinical Medical College of Zhejiang Chinese Medical University, Hangzhou, 310053 China; 5 Academy of Chinese Culture and Health Sciences, Oakland, CA 94612 USA

**Keywords:** Qin-Yu-Qing-Chang decoction, Ulcerative colitis, PPAR-γ, TCA cycle, Facultative anaerobe, *Enterobacteriaceae*

## Abstract

**Background:**

Qin-Yu-Qing-Chang decoction (QYQC), an herbal formula from China, is extensively employed to manage ulcerative colitis (UC) and exhibits potential benefits for colonic function. Nevertheless, the fundamental molecular mechanisms of QYQC remain largely uncharted.

**Methods:**

The primary constituents of QYQC were determined utilizing UHPLC-MS/MS analysis and the effectiveness of QYQC was assessed in a mouse model of colitis induced by dextran sulfate sodium. Evaluations of colon inflammatory responses and mucosal barrier function were thoroughly assessed. RNA sequencing, molecular docking, colonic energy metabolism, and 16S rRNA sequencing analysis were applied to uncover the complex mechanisms of QYQC in treating UC. Detect the signal transduction of the peroxisome proliferator-activated receptor-γ (PPAR-γ) both in the nucleus and cytoplasm. Furthermore, a PPAR-γ antagonist was strategically utilized to confirm the functional targets that QYQC exerts.

**Results:**

Utilizing UHPLC-MS/MS, the principal constituents of the nine traditional Chinese medicinal herbs comprising QYQC were systematically identified. QYQC treatment substantially ameliorated colitis in mice, as evidenced by the improvement in symptoms and the reduction in colonic pathological injuries. Besides, QYQC treatment mitigated the inflammatory response and improved mucosal barrier function. Furthermore, QYQC enhanced the mitochondria citrate cycle (TCA cycle) by triggering PPAR-γ signaling and increasing the proportion of PPAR-γ entering the nucleus. This prevented the unconstrained expansion of facultative anaerobes, particularly pathogenic *Escherichia coli* (*E. coli*, family *Enterobacteriaceae*) and thus improved colitis. Results of molecular docking indicated that the representative chemical components of QYQC including Baicalin, Paeoniflorin, Mollugin, and Imperatorin bound well with PPAR-γ. The impact of QYQC on colitis was diminished in the presence of a PPAR-γ antagonist.

**Conclusions:**

In summary, QYQC ameliorates UC by activating PPAR-γ signaling and increasing the proportion of PPAR-γ entering the nucleus, which enhances the energy metabolism of intestinal epithelial cells and thereby preventing the uncontrolled proliferation of facultative anaerobes.

**Supplementary Information:**

The online version contains supplementary material available at 10.1186/s13020-024-01006-9.

## Background

Ulcerative colitis (UC) typically manifests as recurrent episodes of bloody diarrhea and abdominal pain [1]. In addition to the inflammatory manifestations of the colorectal mucosa, patients with UC are often accompanied by numerous extraintestinal manifestations and complications, affecting not only work efficiency but also mental health [2, 3]. In the preceding years, the accelerated development of various biological agents and small-molecule drugs has brought excellent improvements to patients. However, unavoidable side effects and high costs make these treatments limiting. Worse still, the use of novel drugs has not broken the therapeutic ceiling for UC. Data from induction trials indicate that the remission rates of existing drugs have not exceeded the upper limit of 30% and are far from expectations, given its confusing pathogenesis [4]. Therefore, it is urgent to identify new alternative drugs for treating UC.

Dysbiosis of the gut microbiota, especially the increase in facultative anaerobic bacteria, significantly contributes to the exacerbation of intestinal mucosal inflammation and dysfunction of the intestinal wall [5]. Notably, the characteristic increases in facultative anaerobes, including *Escherichia coli* (*E. coli,* family *Enterobacteriaceae*), often come at the cost of a decrease in obligate anaerobes [6]. Intestinal microbiota analysis findings revealed a significant rise in the prevalence of *E. coli* among individuals with UC [7]. In consequence, suppressing excessive *E. coli* at a relatively low abundance and reconstituting the balance of intestinal flora has become the focus of treatment for UC [8, 9].

Peroxisome proliferator-activated receptor-γ (PPAR-γ), a nuclear receptor, initially resides in the cytoplasm, translocates to the nucleus and interacts with the retinoid X receptor (RXR) upon activation [10]. As a central coordinator of the intestinal inflammation and mucosal barrier, PPAR-γ has been demonstrated to have a broad presence in intestinal epithelial cells [11]. It was reported that PPAR-γ improved cellular metabolism by regulating oxidative phosphorylation (OXPHOS) to activate the mitochondrial citrate cycle (TCA cycle) [12]. Furthermore, research has demonstrated that PPAR-γ signaling influences luminal oxygen availability through reshaping the colonic metabolism toward β-oxidation, which helps prevent the growth of harmful *Escherichia* and *Salmonella* bacteria [13]. Additionally, PPAR-γ negatively regulates the expression and activity of inducible nitric oxide synthase (iNOS), resulting in a substantial decrease in gut nitrate availability [14]. Therefore, PPAR-γ can maintain a low oxygen state in the intestinal epithelium by influencing the homeostatic balance between mitochondrial energy metabolism, including the TCA cycle, OXPHOS, and fatty acid β-oxidation, and the intestinal microbiota, ensuring the predominance of obligate anaerobes in the gut lumen. However, PPAR-γ expression in colonic epithelial cells is notably diminished in UC patients [15, 16]. An increasing number of research studies targeting PPAR-γ as a therapeutic option to combat UC has been implemented, implying its bright prospect [17, 18].

The Chinese herbal medicine Qin-Yu-Qing-Chang decoction (QYQC), which is a modified version of the Qing-Chang-Hua-Shi formula (QCHS) used for treating UC, consists of *Scutellaria baicalensis* Georgi (Huangqin), *Pulsatilla chinensis* (Bunge) Regel (Baitouweng), *Smilax glabra* Roxb. (Tufuling), *Paeonia lactiflora* Pall. (Baishao), *Sanguisorba officinalis* L. (Diyu), *Rubia cordifolia* L. (Qiancao), *Cynanchum paniculatum* (Bunge) Kitag. ex H. Hara (Xuchangqing), *Angelica dahurica* (Hoffm.) Benth. & Hook.f. ex Franch. & Sav. (Baizhi) and *Glycyrrhiza uralensis* Fisch. (Gancao). The plant names have been verified using http://www.theplantlist.org. Detailed information on herbs in QYQC is provided in Table 1. The effectiveness of QCHS has been demonstrated in our previously published randomized clinical trials [19]. We have also conducted experiments in mouse models of colitis to document the pharmacological effects of QCHS and its diverse herbal constituents, which include Huangqin, Baitouweng, Baishao, Diyu, and Baizhi [20, 21]. According to the previous clinical and experimental results, we optimized the composition of QCHS and named it QYQC. Furthermore, due to its proven efficacy, QYQC has been listed as a featured preparation of Jiangsu Province Hospital of Chinese Medicine for the treatment of ulcerative colitis (Y2022zj03). However, the precise functional targets of QYQC in regulating intestinal functions and the underlying mechanisms remain incompletely understood.

**Table 1:** Detailed information on herbs in QYQC

Chinese name	Latin name	Part(s) used	Amount (g)	Place of collection	Batch number
Huangqin	*Scutellaria baicalensis* Georgi	Roots	9	Heibei	230101
Baitouweng	*Pulsatilla chinensis* (Bunge) Regel	Roots	12	Anhui	22121411
Tufuling	*Smilax prolifera* Roxb.	Roots	24	Zhejiang	2022120101
Baishao	*Paeonia lactiflora* Pall.	Roots	9	Zhejiang	230201
Diyu	*Sanguisorba officinalis* L.	Roots	12	Jiangsu	230401
Qiancao	*Rubia cordifolia* L.	Roots and rhizomes	9	Anhui	22122710
Xuchangqing	*Cynanchum paniculatum* (Bunge) Kitag. ex H.Hara	Roots and rhizomes	12	Shandong	22120608
Baizhi	*Angelica dahurica* (Hoffm.) Benth. & Hook.f. ex Franch. & Sav.	Roots	6	Sichuan	2022110101
Gancao	*Glycyrrhiza uralensis* Fisch.	Roots and rhizomes	3	Neimenggu	230102

The present study proved that QYQC markedly alleviated disease severity, enhanced mucosal barrier functions, and ameliorated microbiota dysbiosis. The underlying mechanism involved optimizing the energy metabolism in mitochondria and inhibiting the excessive growth of facultative anaerobes, particularly *E. coli*, by stimulating PPAR-γ signaling and enhancing the proportion of PPAR-γ translocating into the nucleus.

## Materials and methods

### Reagents

Dextran sulfate sodium (DSS) (MW: 36,000–50,000 kDa, CAS: 9011-18-1) was purchased from MP Biomedicals (California, USA). 5-Aminosalicylic acid (5-ASA, #A79809) was bought from Sigma-Aldrich (St. Louis, MO, USA). T0070907 (#HY-13202) was provided by MCE (Shanghai, China). HiScript® III RT SuperMix (#R323-01), ChamQ SYBR Master Mix for qPCR (#Q311-02/03) and RNA isolation kit (#R711) were purchased from Vazyme Biotech (Nanjing, Jiangsu, China). Nuclear and Cytoplasmic Protein Extraction Kit was bought from Beyotime Biotechnology (Shanghai, China). For western blotting and immunofluorescence, primary antibodies against PPAR-γ (#2435) and iNOS (#13120) were brought from Cell Signaling Technology (Danvers, MA, USA). Claudin-4 (#ab15104) was bought from Abcam (Cambridge, UK). Zonula occludens (ZO-1, #GB111981) and Mucin2 (MUC2, #GB14110) were provided by Servicebio Biotech (Wuhan, Hubei, China). Histone-H3 (#17168-1-AP) and Beta Actin (#66009-1-Ig) were obtained from Proteintech Group (Rosemont, USA). Interleukin (IL)-1β (#EK201B), IL-6 (#EK206), Tumor necrosis factor-α (TNF-α, #EK282) and IL-10 (#EK210) ELISA kits were gotten from Multi Sciences (Hangzhou, Zhejiang, China). Lipopolysaccharides (LPS) ELISA Kit (#ml002005) was purchased from Mlbio (Shanghai, China).

### Preparation and quality control of QYQC

All herbs used in QYQC were provided by Affiliated Hospital of Nanjing University of Chinese Medicine. Huangqin, Baitouweng, Tufuling, Baishao, Diyu, Qiancao, Xuchangqing, Baizhi, and Gancao were blended in the following proportions: 3:4:8:3:4:3:4:2:1 (total 96 g). Herbs were soaked in 960 mL of double distilled water for 30 min, boiled for 40 min, and the herbal liquid was collected. Subsequently, this step was repeated once, and all the herbal liquids were combined before being subjected to rotary evaporation. Eventually, the liquid was condensed to 132 mL for QYQC-L group or 66 mL for QYQC-H group.

Detailed methods of UHPLC-MS/MS analysis, including the quantification methods of the markers, the conditions of multiple reaction monitoring (MRM), and the extracted MRM chromatograms with PubChem CID in the standards and QYQC are displayed in Additional file 1: Table S1-2 and Fig S1A-B.

### Animal experiments and treatments

Six to eight-week-old male C57BL/6J mice (Zhejiang Vital River Laboratory Animal Technology Co., Ltd., Zhejiang, China) were housed in a specific-pathogen-free facility (Animal usage license number: SYXK(苏)2022-0070). Animal experiments were performed according to the Guidelines for Animal Experimentation of the Nanjing University of Chinese Medicine. The Animal Ethics Committee of Affiliated Hospital of Nanjing University of Chinese Medicine approved the animal experiments (Application Number: 2022DW-13-01).

Following a one-week adaptation, mice were randomly assigned to different treatment groups. Colitis was induced by administering 2.5% DSS for a duration of 7 days. Different doses of QYQC (7.3, 14.6 g/kg/day), 5-ASA (100 mg/kg/day), T0070907 (5 mg/kg/day), or distilled water were selected and orally administered via gavage from day 1 to day 10 according to the experimental requirements [22]. The disease activity indexes (DAI) were evaluated by calculating the mean score of body weight loss, stool consistency, and the extent of blood in feces as previously described [23].

### Histopathological assessment

Upon removal, the distal colons were fixed using 4% paraformaldehyde and subsequently embedded in paraffin. Sections, 4 μm thick, were cut from the embedded colon tissue for further staining. Staining procedures, including Hematoxylin and Eosin (H&E) as well as Alcian Blue (AB)/Periodic Acid-Schiff (PAS) staining, were performed using commercially available kits. A comprehensive histopathological score was comprehensively assessed based on previous reports [24].

### Western blotting

The proteins were isolated with RIPA buffer. Subsequently, proteins were separated and transferred onto PVDF membranes and incubated overnight with appropriate primary antibodies targeting PPAR-γ (1:1000), iNOS (1:1000), Claudin-4 (1:1000), and β-actin (1:5000). The grayscale values were then observed by the Bio-Rad GelDoc XR+system. Finally, Image J was applied for grayscale value analysis.

### Immunofluorescence

For immunofluorescence staining, colonic sections were subjected to overnight incubation at 4 °C with polyclonal antibodies against ZO-1 and MUC2. On the following day, the corresponding fluorescent anti-rabbit or anti-mouse secondary antibody were added dropwise. Subsequently, they were incubated at room temperature without light for 1 h. DAPI was incubated under the same conditions for 10 min. Images were acquired after adding anti-fluorescence quenching reagent.

### Enzyme-linked immunosorbent assay (ELISA)

Colonic tissue was homogenized and lysed in PBS at 4 °C. After centrifugation for 10 min, the resulting supernatants were used to measure the level of cytokines (IL-1β, IL-6, TNF-α, IL-10) and LPS, observing the manufacturer’s guidance.

### Quantitative real-time PCR

Total RNA was extracted using an RNA isolation kit and then reverse transcribed into cDNA in accordance with the manufacturer’s guidelines. Quantitative real-time PCR analysis was accomplished using ChamQ SYBR Master Mix for qPCR and analyzed by LightCycler® 96 PCR system (Roche). The primer sequences are provided in Additional file 2.

### RNA sequencing

The detailed method of RNA sequencing is presented in Additional file 3.

### Molecular docking

The representative chemical components of QYQC analyzed by UHPLC-MS/MS were selected for molecular docking. The 3D conformer of the chemical compounds was downloaded from PubChem (https://pubchem.ncbi.nlm.nih.gov/) and subsequently converted to Mol2 format using OpenBabel (v2.4.1). The 3D structure of PPAR-γ (PDB ID: 6MS7) were obtained from RCSB Protein Data Bank (https://www.rcsb.org/). Pymol (v2.5.7) was used for molecular pre-processing. AutoDockTools (v1.5.6) was adopted for docking PPAR-γ with different ligands. The conformation with the lowest intermol energy was recorded and used for further analysis. The visualization processing of the results was carried out in Pymol and LigPlus.

### Gut microbiota analysis

The detailed method of 16S rRNA sequencing is presented in Additional file 4.

### Bactericidal assay

Collected fresh mouse feces and formulated a 10 mg/mL suspension with sterile PBS. The resuspended mixture was spread onto LB agar plates and then placed in an anaerobic incubator at under the following conditions: 37 °C, 80% N_2_, 10% H_2_, and 10% CO_2_. The colony-forming units (CFUs) on the agar plates were quantified after incubation periods of 12 and 24 h.

### Energy metabolomic analysis

10 mg of colon tissue was weighed, and each mixture was homogenized for 3 min after adding 20 µL of deionized water and 120 µL of pre-cooled methanol solution. The mixtures were then centrifuged at 18000×*g* for 15 min at 4 °C. Equal volumes of 200 mM 3-NPH and 120 mM EDC were added to 20 µL of the supernatant. After a 60 min reaction at 30 °C and 1450 rpm, the mixture was diluted with 350 µL of ice-cold methanol solution. Subsequently, 150 µL of the supernatant was transferred for injection after centrifugation at 4 °C and 4000×*g* for 20 min.

The experiment followed the method provided by Metabo-Profile Biotechnology Co., Ltd (Shanghai, China). Detailed procedures for energy metabolomic analysis are provided in Additional file 5.

### Statistical analysis

All data were presented as means ± SEM and analyzed using GraphPad Prism 8.0 software. One-way ANOVA was performed to analyze the statistical difference. Nonparametric statistical tests (Wilcoxon tests) were used in cases where the data did not adhere to a normal distribution. Statistical significance was defined as *p* < 0.05.

## Results

### UHPLC-MS/MS analysis of QYQC

**Fig. 1:**
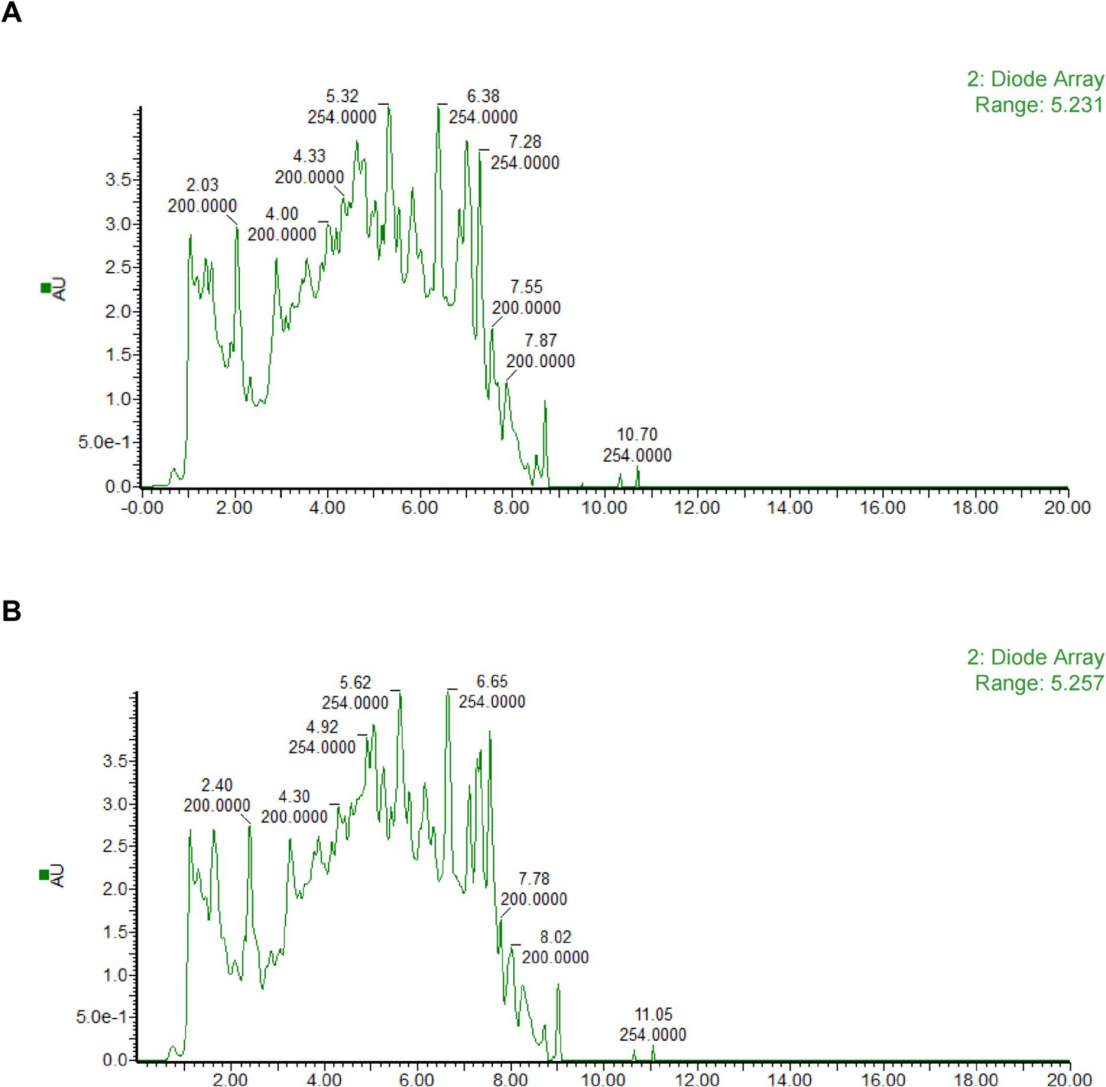
UHPLC-MS/MS analysis of QYQC components. Base peak chromatograms (BPC) of QYQC in both **A** positive mode and **B** negative mode

The key chemical constituents of QYQC were identified using UHPLC-MS/MS analysis and visualized in the base peak chromatograms (BPC) obtained through both positive ESI+ (Fig. 1A) and negative ESI− (Fig. 1B) modes. The concentration of prominent compounds from each herb identified in QYQC were annotated and presented in Additional file 1: Table S3.

### QYQC alleviates DSS-induced colitis in mice

**Fig. 2:**
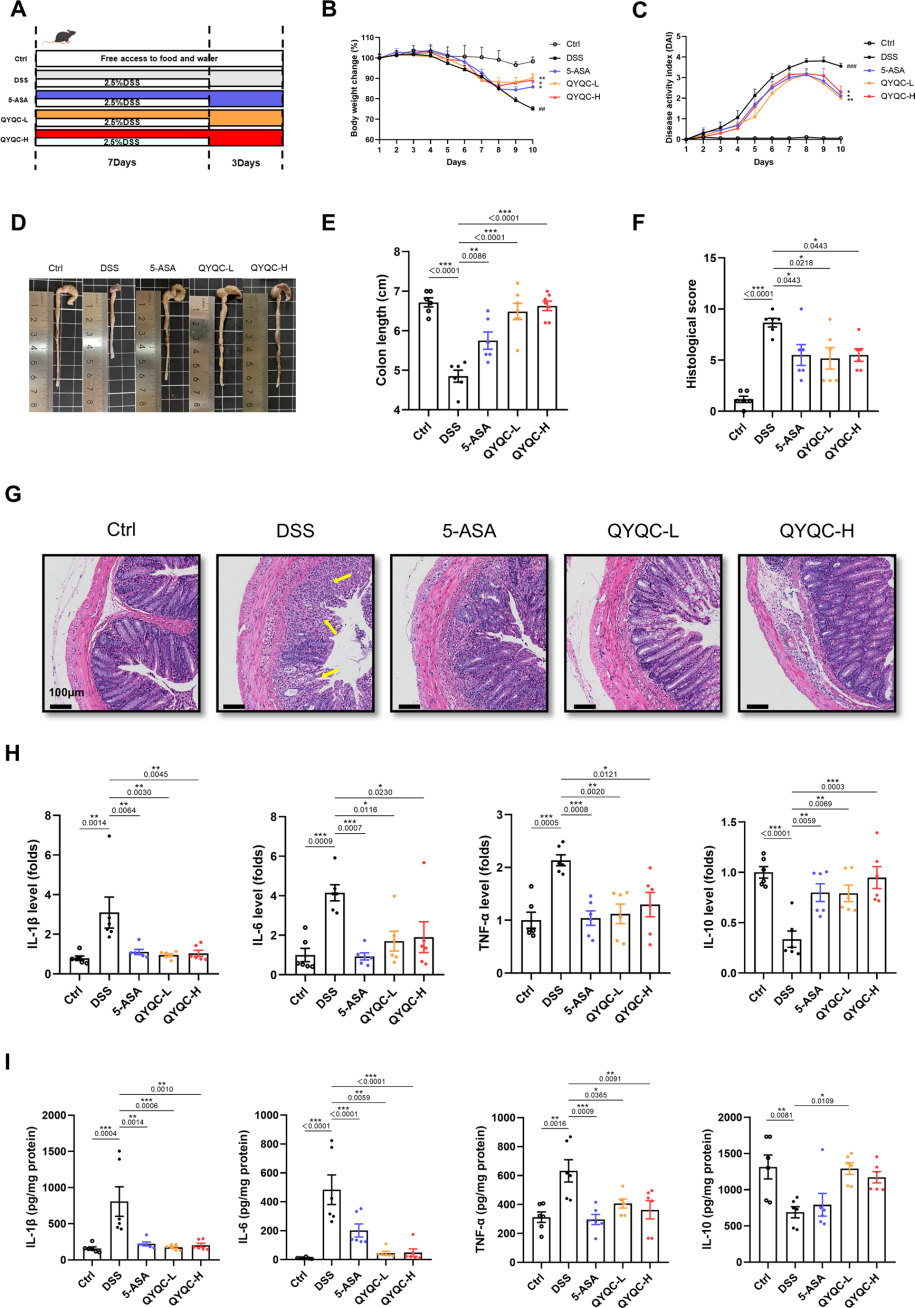
QYQC alleviates DSS-induced colitis in mice. **A** Study flow chart. **B** Body weight change throughout the study. **C** DAI scores. **D**, **E** Colon length of each group. **F** Histological score. **G** H&E-stained colonic sections (scar bar, 100 µm). The arrows indicate the pathological damage. **H**, **I** Relative mRNA expression and protein levels of IL-1β, IL-6, TNF-α, and IL-10 in colon tissue. Data are displayed as Mean ± SEM (*n* = 6–7). *p < 0.05, **p < 0.01, ***p < 0.001

To investigate the ameliorative impact of QYQC on DSS-induced colitis, we initiated experimental colitis in mice by subjecting them to a 7-day regimen of 2.5% DSS, then allowed a subsequent 3-day period of fresh water for recovery (Fig. 2A). In contrast to the DSS group, both QYQC and 5-ASA treatment exhibited significant alleviation of DSS-induced colitis, as evidenced by substantial reductions in body weight loss, decreased DAI, and mitigation of colon shortening (Fig. 2B–E). Histological analysis according to H&E-staining further showed obvious pathological damage in DSS group. Nonetheless, QYQC and 5-ASA treatment conspicuously mitigated the pathological damage to colon tissue, encompassing factors such as inflammatory cell infiltration, crypt loss, and mucosal injury (Fig. 2F, G). As inflammatory cytokines are crucial in the initiation of colitis, we assessed the influence of QYQC and 5-ASA on these molecules. Notably, treatment with QYQC and 5-ASA led to a substantial reduction in the mRNA and protein levels of IL-6, IL-1β, and TNF-α, while concurrently increasing the levels of IL-10 in colonic tissue (Fig. 2H, I).

### QYQC reinstates the intestinal barrier in mice with colitis

**Fig. 3:**
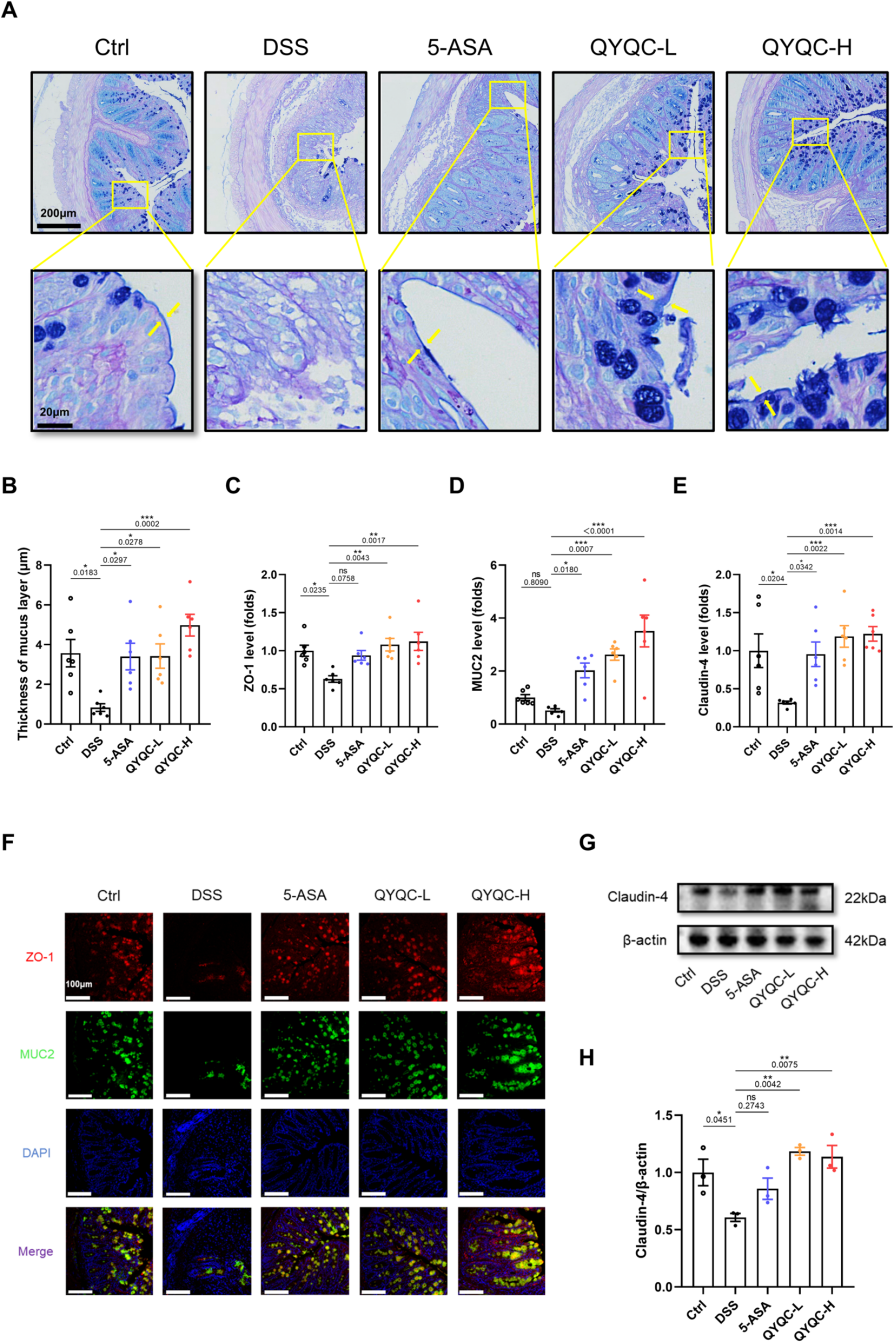
QYQC reinstates the intestinal barrier in mice with colitis. **A** AB/PAS-stained colonic sections (scar bar, 200 µm, 20 µm). The arrows indicate the mucus layer. **B** Mucus thickness (*n* = 6). **C**–**E** Relative mRNA expression of ZO-1, MUC2, and Claudin-4 in colon tissue (*n* = 6). **F** Immunofluorescent analysis of ZO-1 and MUC2 in colon section (scar bar, 100 µm). **G**, **H** Examination of Claudin-4 protein expression in colon tissue using immunoblotting (*n* = 3). Data are displayed as Mean ± SEM. *p < 0.05, **p < 0.01, ***p < 0.001

As depicted in Fig. 3A, we next examined the influence of QYQC on the colonic mucosal barrier using AB/PAS staining. In the DSS group, there were fewer goblet cells producing mucus, resulting in a thinner mucosal layer. However, QYQC and 5-ASA treatment attenuated such damage to normal (Fig. 3B). ZO-1, MUC2, and Claudin-4, which are critical for gut homeostasis, are key components of tight junction proteins and the mucus layer. The mRNA and protein expression of ZO-1, MUC2, and Claudin-4 were downregulated in DSS group, while QYQC and 5-ASA treatment significantly upregulated the expression of ZO-1, MUC2, and Claudin-4 (Fig. 3C–H).

### QYQC activates the PPAR-γ signaling pathway

**Fig. 4:**
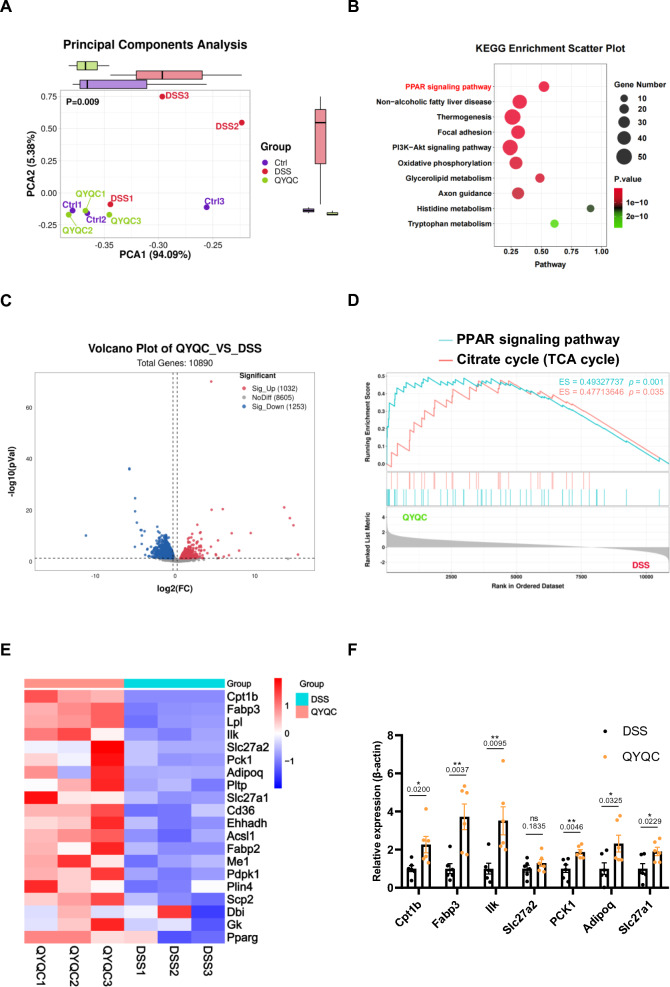
QYQC activates the PPAR-γ signaling pathway. **A** PCA analysis of transcriptional profiles from mouse colonic tissues (*n* = 3). **B** KEGG pathway enrichment analysis among mice in the QYQC, DSS, and Ctrl groups (*n* = 3). **C** Volcano plot of differentially expressed genes between QYQC and DSS groups (*n* = 3). **D** GSEA of PPAR signaling pathway and TCA cycle between QYQC and DSS groups (*n* = 3). **E** Heat map of differentially expressed genes in PPAR signaling pathway (fold change > 1.2 and *p* < 0.05). **F** Relative mRNA expression of differentially expressed genes in PPAR signaling pathway (*n* = 6). Data are displayed as Mean ± SEM. *p < 0.05, **p < 0.01

To gain a deeper understanding of how QYQC alleviates colonic inflammation and enhances mucosal barrier function, we conducted comprehensive transcriptome profiling of colonic tissues using RNA sequencing. The transcriptomes were significantly different among Ctrl, DSS, and QYQC groups (Fig. 4A). Notably, the PPAR signaling pathway emerged as one of the most significantly enriched functional pathways (Fig. 4B). By applying stringent criteria for significance (fold change > 1.2 and *p* < 0.05), we identified a total of 2285 altered genes, with 1032 genes upregulated and 1253 genes downregulated in the colons of QYQC group compared to the DSS group (Fig. 4C). Gene set enrichment analysis (GSEA) revealed that PPAR signaling pathway and TCA cycle were suppressed in the DSS group compared to the Ctrl group (Additional file 6: Fig. S2). Remarkably, it was reactivated by QYQC treatment (Fig. 4D, E). Coincidentally, we confirmed the mRNA expression of the most significant differential genes via quantitative real-time PCR (Fig. 4F).

**Fig. 5:**
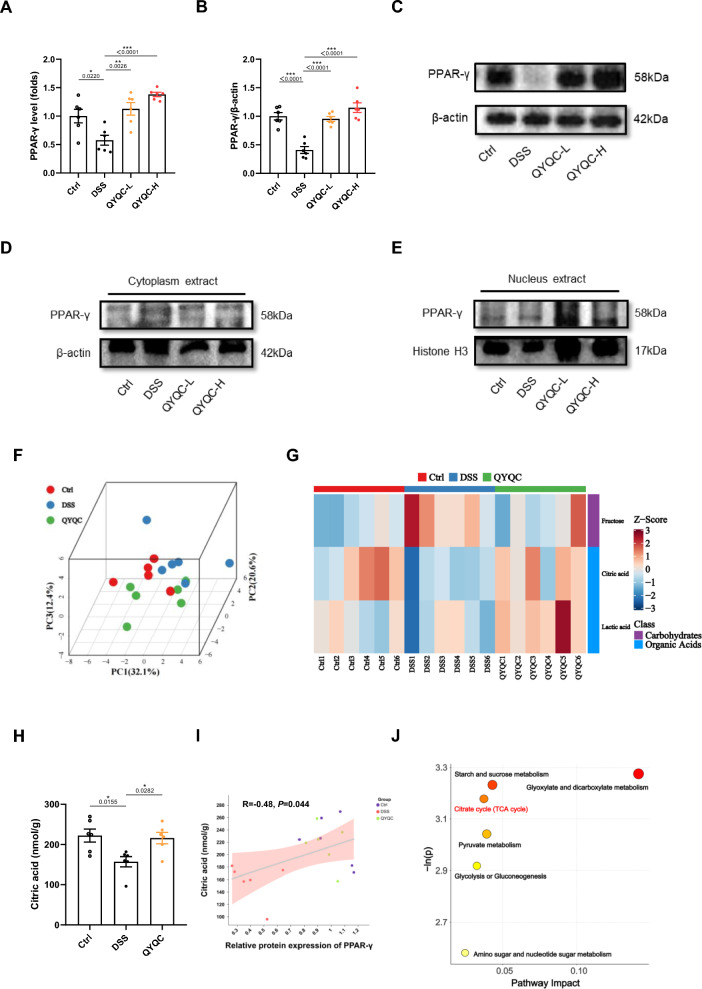
QYQC regulates PPAR-γ to improve colonocyte energy metabolism in mice. **A** Relative mRNA (*n* = 6) and **B**, **C** protein expression of PPAR-γ in colon tissue (*n* = 3). **D** The protein expression of PPAR-γ in the cytoplasm and **E** nucleus extract (*n* = 3). **F** PCA analysis. **G** Heatmap of potential biomarkers (VIP > 1, |log2FC|≥ 0 and *p* < 0.05). **H** Citric acid levels in colonic tissue. **I** Spearman correlation analysis between citric acid levels and the protein expression of PPAR-γ. **J** KEGG pathway analysis. Data are displayed as Mean ± SEM. *p < 0.05, **p < 0.01, ***p < 0.001

Patients with UC frequently demonstrate a deficiency in PPAR-γ within intestinal epithelial cells. Interestingly, we observed epithelial expression inhibition of PPAR-γ at the mRNA level in DSS group (Fig. 5A). This unfavorable misalignment was reversed after QYQC treatment and the protein level of PPAR-γ was further confirmed (Fig. 5B, C). As a nuclear receptor, PPAR-γ regulates gene expression associated with inflammation control within the nucleus [25]. Our findings indicated no significant changes in cytoplasmic PPAR-γ levels across the experimental groups (Fig. 5D). Notably, the protein level of PPAR-γ within the nucleus exhibited a substantial increase in QYQC group (Fig. 5E). Results of molecular docking indicated that the representative chemical components of QYQC including Baicalin, Paeoniflorin, Mollugin, and Imperatorin bound well with PPAR-γ. These four components may be key ingredients in treating UC. The detailed information is displayed in Additional file 6: Fig. S3 and Table S4.

### QYQC regulates PPAR-γ to improve colonocyte energy metabolism in mice

Enhancing PPAR-γ activity improves mitochondrial OXPHOS and the TCA cycle, inhibiting the pathological expansion in facultative anaerobes and ameliorating colitis [12]. After confirming the activating effect of QYQC on the PPAR signaling in colonic epithelial cells of mice with colitis, we proceeded to conduct targeted quantitative assessments of colonic energy metabolism. PCA analysis demonstrated a distinct separation between the DSS group and the Ctrl as well as QYQC groups (Fig. 5F). Under the conditions of VIP > 1, |log2FC| ≥ 0 and *p* < 0.05, lactic acid, fructose, and citric acid were identified as potential biomarkers (Fig. 5G). In the colonic tissues of the DSS group, there was a significant reduction in citric acid levels compared to the Ctrl group, and QYQC effectively reversed this decline (Fig. 5H). Furthermore, the colonic tissue citric acid levels were significantly positively correlated with the protein expression of PPAR-γ (Fig. 5I). The pathway analysis results involving potential biomarkers demonstrated that the intervention of QYQC significantly impacts cellular energy metabolism pathways, encompassing the TCA cycle, glyoxylate and dicarboxylate metabolism, as well as starch and sucrose metabolism (Fig. 5J).

### QYQC ameliorates gut microbiota dysbiosis of colitis mice

**Fig. 6:**
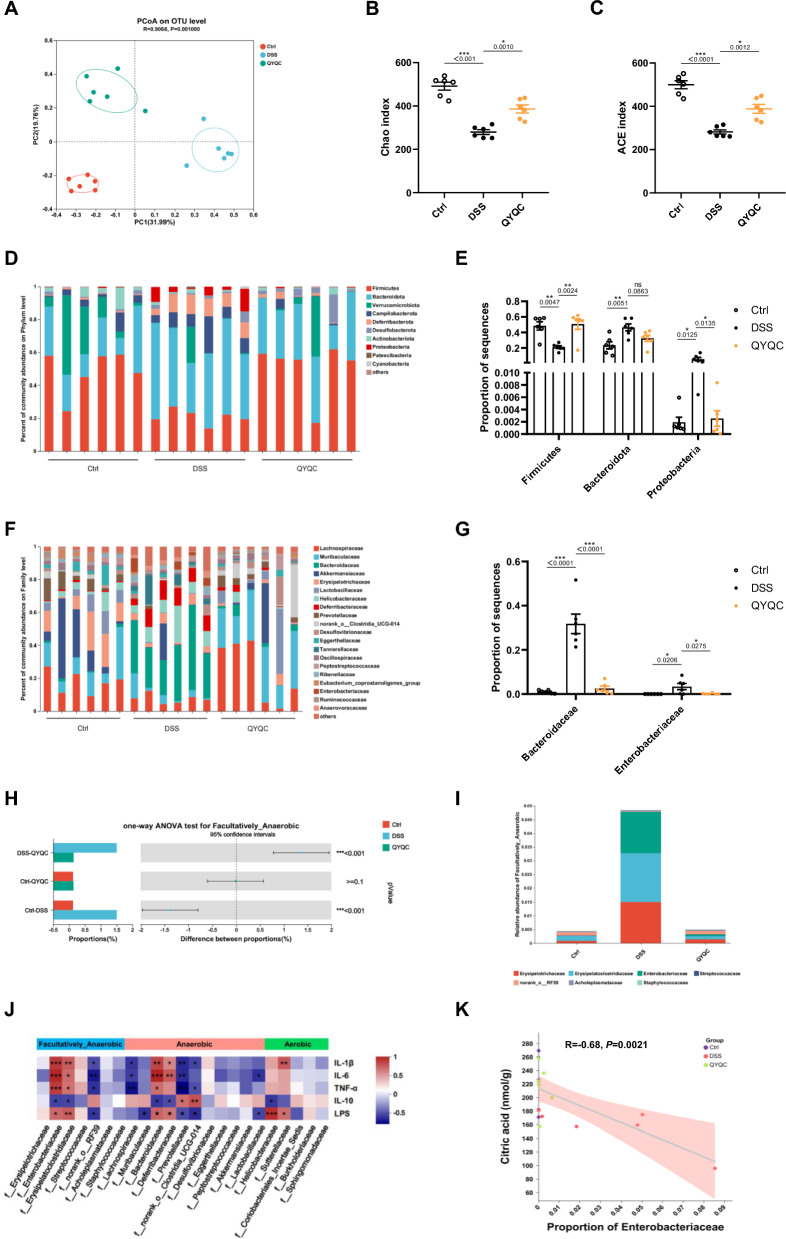
QYQC ameliorates gut microbiota dysbiosis of colitis mice. **A** PCoA plotting. **B**, **C** Alpha diversity analysis (Chao and ACE index) from different groups. **D**, **E** Composition of gut microbial taxa at the phylum level and **F**, **G** at the family level. **H** one-way ANOVA test for facultative anaerobic bacteria in Ctrl, DSS and QYQC groups. **I** Relative abundance and composition of facultative anaerobic bacteria by BugBase phenotypic predictive analysis at the family level. **J** Spearman correlation analysis between intestinal microbiota and anti-inflammatory factors or LPS in colon tissue among different groups. **K** Spearman correlation analysis between the *Enterobacteriaceae* abundance and the level of citric acid. Data are expressed as Mean ± SEM (*n* = 6). *p < 0.05, **p < 0.01, ***p < 0.001

The disruption of gut microbiome homeostasis is pivotal in UC pathogenesis. Metabolic pathways such as the mitochondrial TCA cycle, are regulated by the PPAR-γ signaling pathway, which inhibits the proliferation of gut luminal facultative anaerobes by improving the energy metabolism level of intestinal epithelial cells [13]. To gain deeper insights into the influence of colonic epithelial cell metabolism on the gut microbiota, we detected disparities in the construction of the gut microbiota and found significant separation after QYQC treatment. As is shown in Fig. 6A by Principal coordinates analysis (PCoA), the gut microbiota composition in DSS group has been changed significantly, which was different from that of the Ctrl group and the QYQC group. Notably, QYQC administration upregulated the alpha diversity indicators including the Chao index and ACE index manifested a remarkable decrease in DSS group (Fig. 6B, C). Furthermore, we analyzed the composition of gut microbial taxa. At the phylum level, compared to the Ctrl group, the DSS group exhibited decreased Firmicutes abundance and notable increases in Bacteroidota and Proteobacteria abundances. (Fig. 6D, E). At the family level, QYQC treatment significantly lowered the abundance of *Bacteroidaceae* and *Enterobacteriaceae* (Fig. 6F, G). QYQC treatment also effectively restrained the expansion of *E. coli* (Additional file 6: Fig. S4A-B) and mitigated the production of LPS (Additional file 6: Fig. S4C).

An imbalance in gut bacteria characterized by a decrease in obligate anaerobes and uncontrolled growth of facultative anaerobes like *E. coli* is one of the typical features of UC [6]. Interestingly, the proportion of facultative anaerobes in DSS group was remarkably increased, which was downregulated by QYQC treatment (Fig. 6H). The results of species-phenotypic contribution analysis at family level showed that *Erysipelatoclostridiaceae, Erysipelotrichaceae, Enterobacteriaceae, norank_o__RF39, Acholeplasmataceae, Streptococcaceae, and Staphylococcaceae* were the most abundant facultative anaerobes (Fig. 6I). Interestingly, correlation analysis revealed that facultative anaerobes such as *Enterobacteriaceae* and *Erysipelatoclostridiaceae* had a markedly favorable correlation with the level of LPS, IL-1β, IL-6 and TNF-α, and an obvious negative correlation with IL-10 in the colon. In contrast, obligate anaerobes like *Lachnospiraceae* were conspicuously negatively correlated with IL-1β, IL-6, and TNF-α. *Prevotellaceae* and *Clostridia_UCG-014* were significantly negatively correlated with IL-1β, IL-6, TNF-α, LPS, and positively correlated with IL-10. Furthermore, *Lactobacillaceae* was significantly negatively correlated with IL-6 and LPS (Fig. 6J). Notably, *Enterobacteriaceae* was negatively correlated with the level of citric acid in colon tissue (Fig. 6K).

Next, we analyzed the relationship between gut microbiota abundance at genus level and colonic metabolites associated with energy metabolism. Facultative anaerobes (*Escherichia-Shigella* and *Clostridium_innocuum_group*) and aerobics represented by *Novosphingobium* revealed a significant negative correlation with citric acid levels in colonic tissues. Meanwhile, obligate anaerobes (*Lactobacillus*, *Enterorhabdus*, *Adlercreutzia* and *Candidatus_Saccharimonas*) exhibited a notable positive correlation with the level of citric acid. Furthermore, obligate anaerobes represented by *Desulfovibrio* and *Akkermansia* were negatively correlated with the level of fumaric acid (Additional file 6: Fig. S5).

Inhibition of the PPAR-γ signaling pathway suppresses the mitochondrial TCA cycle and OXPHOS in intestinal epithelial cells, leading to a decrease in bioavailability restriction of oxygen in the colonic lumen, which results in the dysbiotic expansion of facultative anaerobes, accompanied by increased synthesis of iNOS [10, 26]. Coincidentally, we observed an upregulation of iNOS expression in the DSS group, while QYQC inhibited the overexpression of iNOS (Additional file 6: Fig. S6).

### The impact of QYQC on colitis is diminished in the presence of a PPAR-γ antagonist

**Fig. 7:**
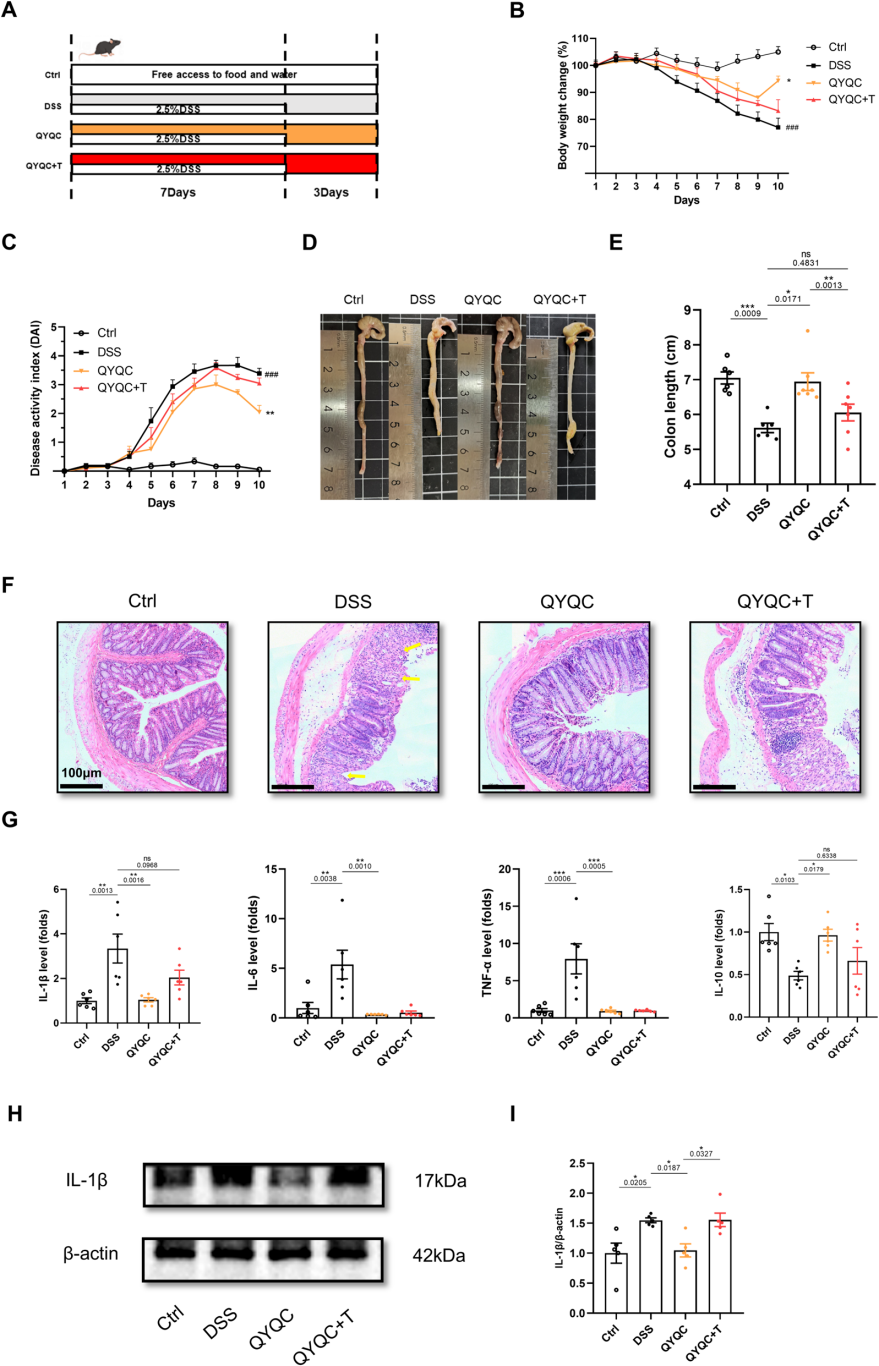
The impact of QYQC on colitis is diminished in the presence of a PPAR-γ antagonist. **A** Study flow chart. **B** Body weight change throughout the study (*n* = 6–7). **C** DAI scores (*n* = 6–7). **D**, **E** Colon length (*n* = 6–7). **F** H&E-stained colonic sections (scar bar, 100 µm). The arrows indicate the pathological damage. **G** Relative mRNA expression of IL-1β, IL-6, TNF-α, and IL-10 in colon tissue (*n* = 6). **H**, **I** The protein expression of IL-1β in colon tissue examined by immunoblotting (*n* = 5). Data are displayed as Mean ± SEM. *p < 0.05, **p < 0.01, ***p < 0.001

PPAR-γ signaling pathway is indispensable in terms of controlling intestinal homeostasis. To verify the association between PPAR-γ signaling activation and improved intestinal inflammation, we administered the PPAR-γ signaling antagonist T0070907 to mice with DSS-induced colitis through daily oral gavage (Fig. 7A). As expected, QYQC treatment shielded colitis mice from body weight loss, increased DAI score and shortened colon length. However, the protective effects of QYQC on colitis were nullified upon the inhibition of PPAR-γ signaling (Fig. 7B–E). Moreover, the maintenance effect of QYQC on colonic epithelial structure was also abolished in QYQC+T group (Fig. 7F). Additionally, the application of T0070907 upregulated IL-1β mRNA expression and downregulated IL-10 mRNA levels (Fig. 7G). The protein expression of IL-1β was significantly elevated in QYQC+T group compared with QYQC group (Fig. 7H, I).

**Fig. 8:**
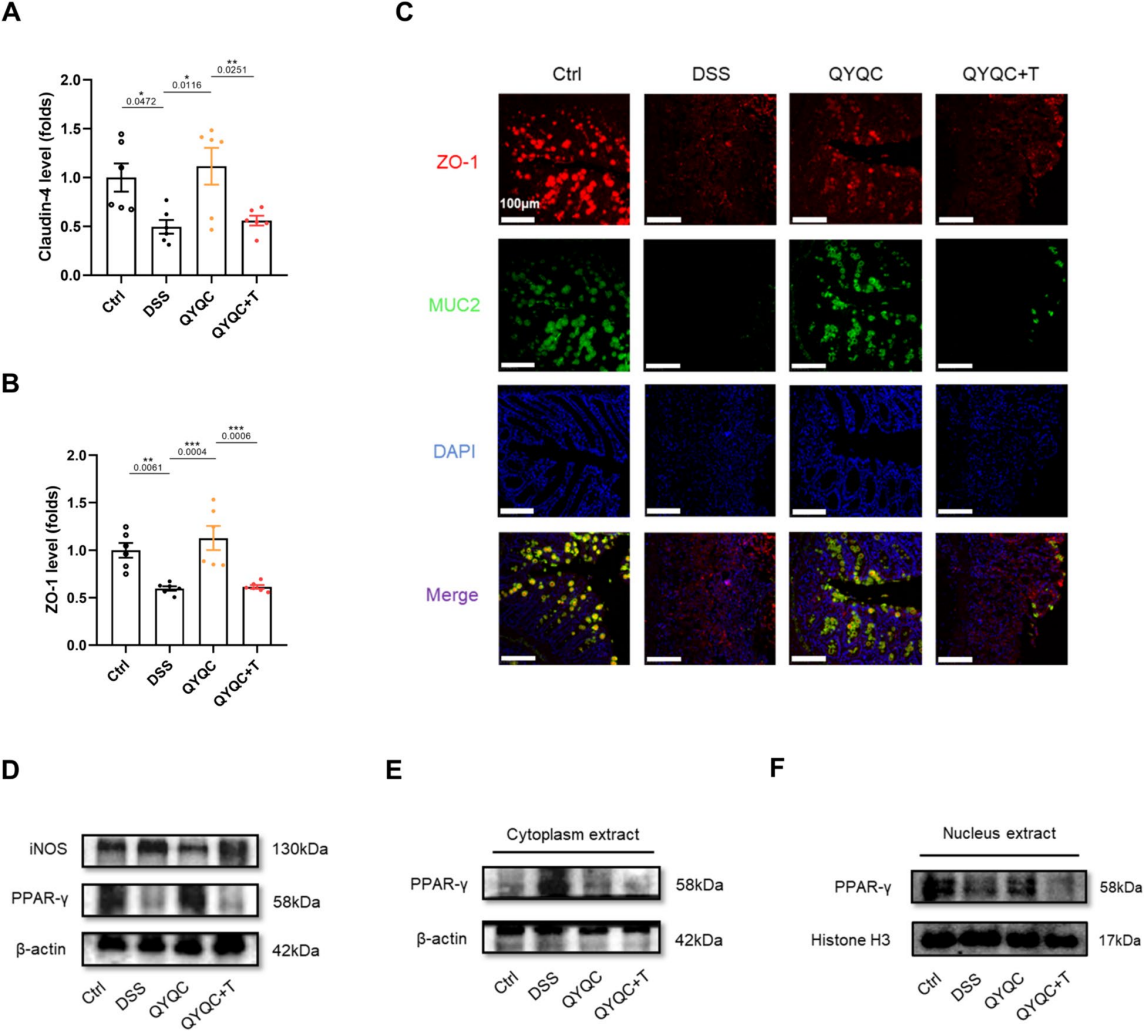
The effect of QYQC on intestinal barrier is diminished by PPAR-γ antagonist. **A**, **B** Relative mRNA expression of Claudin-4 and ZO-1 in colon tissue (*n* = 6). **C** Immunofluorescent analysis of ZO-1 and MUC2 in colon section (scar bar, 100 µm). **D** The protein expression of PPAR-γ in colon tissue examined by immunoblotting (*n* = 3). **E** The protein expression of PPAR-γ in cytoplasm and **F** in nucleus extract examined by immunoblotting (*n* = 3). Data are displayed as Mean ± SEM. *p < 0.05, **p < 0.01, ***p < 0.001

Coincidentally, the positive impact of QYQC on the mucosal health of mice with colitis was similarly hindered by a PPAR-γ inhibitor. The mRNA expression of Claudin-4 and ZO-1 were significantly decreased in QYQC+T group compared with QYQC group (Fig. 8A, B). Furthermore, the protein expression of ZO-1 and MUC2 in QYQC+T group were also downregulated in comparison with QYQC group (Fig. 8C). Additionally, the protein expression of PPAR-γ in colon tissue was decreased in both the DSS and QYQC+T groups (Fig. 8D). It was also inhibited both in the cytoplasm and in the nucleus by PPAR-γ antagonist T0070907 (Fig. 8E, F). On the contrary, the protein expression of iNOS was upregulated in DSS and QYQC+T group, which was reduced by QYQC treatment (Fig. 8D).

**Fig. 9:**
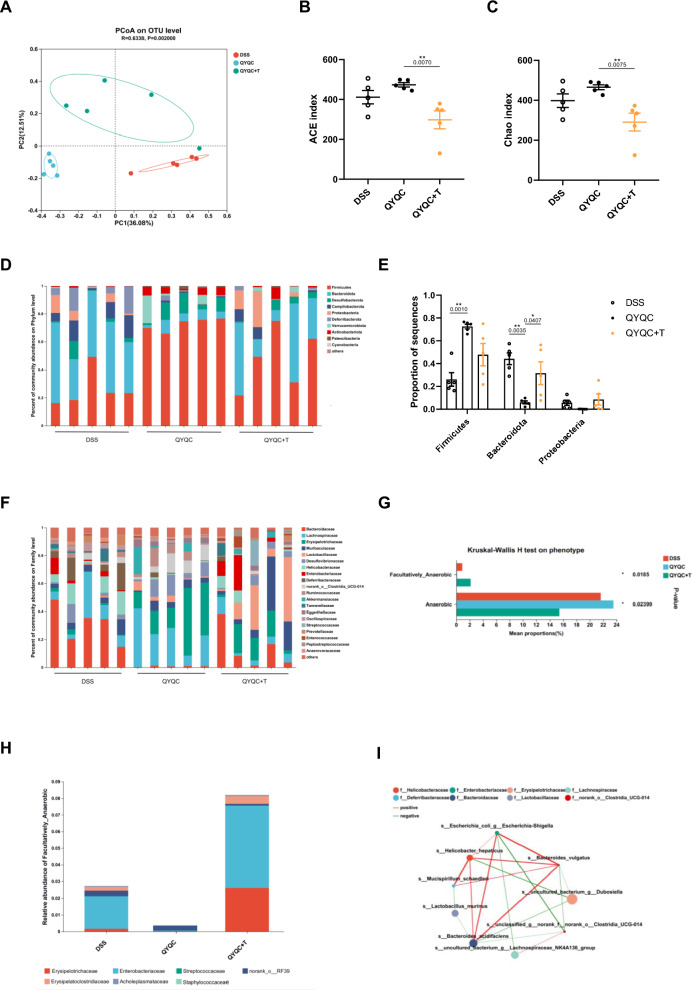
The effect of QYQC on potentially pathogenic microbiota is reversed by PPAR-γ antagonist. **A** PCoA plotting (*n* = 5). **B**, **C** Alpha diversity analysis (Chao and ACE index) from different groups (*n* = 5). **D**, **E** Composition of gut microbial taxa at the phylum and **F** family level (*n* = 5). **G** Mean proportions of facultative anaerobes and obligate anaerobes among different groups (*n* = 5). **H** Relative abundance and composition of facultative anaerobes (*n* = 5). **I** Single factor network analysis of the mice gut microbiota. Species with absolute values of the correlation coefficient > 0.5 and *p* < 0.05 were displayed. The node size is proportionate to the overall abundance, and node colors represent different family classifications. Line colors indicate positive (*red*) and negative (*green*) correlations, with line thickness reflecting the strength of the correlation coefficient (*n* = 5). Data are expressed as Mean ± SEM. *p < 0.05, **p < 0.01, ***p < 0.001

Finally, to unravel the pivotal role of PPAR-γ signaling in the complex interactions between microbiota and colon Inflammation. We examined the intestinal microbial composition of the DSS, QYQC, and QYQC+T group. The inhibition of PPAR-γ signaling relieved the beneficial impact of QYQC on gut microbial construction (Fig. 9A). The ACE and Chao indices were also remarkably decreased in QYQC+T group (Fig. 9B, C). The relative abundance of Bacteroidota notably increased after PPAR-γ signaling inhibition (Fig. 9D, E). Furthermore, the relative abundance of facultative anaerobes decreased in QYQC group but increased after T0070907 treatment at the family level (Fig. 9F–H). Conversely, the relative abundance of obligate anaerobes increased in QYQC group but decreased in QYQC+T group (Fig. 9F–H). BugBase phenotypic predictive analysis showed that the main components of facultative anaerobes including *Enterobacteriaceae, Erysipelotrichaceae, norank_o__RF39, Erysipelatoclostridiaceae, Erysipelatoclostridiaceae, Streptococcaceae* and *Staphylococcaceae* (Fig. 9I).

At the genus level, Single-factor network analysis highlighted *E. coli* (facultative anaerobes) as one of the most significant cordings in the network and was significantly negatively correlated with *Clostridia_UCG-014* and *Lachnospiraceae_NK4A136_group* (obligate anaerobes) (Fig. 9I). As a representative of facultative anaerobic bacteria, the exceptional expansion of *E. coli* was significantly restrained by QYQC treatment (Additional file 6: Fig. S7A-B). Moreover, the application of the PPAR-γ antagonist not only reversed the limitation imposed by QYQC but also significantly increased the abundance of LPS in colonic tissue (Additional file 6: Fig. S7C).

## Discussion

UC involves complex interactions between gut microbiome and intestinal epithelial cells, affecting mucosal integrity and immune activation [27, 28]. Reduced PPAR-γ expression in colonic tissue was observed in individuals with UC and colitis mice [29, 30]. Although numerous drugs such as mesalazine and thiazolidinediones (TZDs) have been proven to relieve UC by agitating PPAR-γ, issues including drug side effects and safety limit their application [31]. Therefore, there is a pressing demand for the creation of potent agents.

Growing and more compelling research evidence indicates that Traditional Chinese Medicine exhibits distinct benefits in treating UC [32, 33]. QYQC is an effective Chinese medicinal modification from QCHS to alleviate UC [19]. In this study, we found that QYQC promotes mucosal integrity and addressed gut microbiota dysbiosis in experimental colitis mice. Meanwhile, our study confirmed that the protective effect was achieved through the PPAR-γ signaling-mediated mitochondria-microbiota crosstalk, thereby inhibiting the proliferation of facultative anaerobes (notably *E. coli*) in the intestine.

Excessive colorectal inflammatory response and disruption of intestinal mucosal integrity are typical features of UC [34]. In this study, mice treated with QYQC suffered milder intestinal inflammation, manifested by lighter colon pathological damage. More importantly, QYQC demonstrated excellent anti-inflammatory effects by reducing pro-inflammatory cytokines (IL-1β, IL-6, and TNF-α) and increasing the level of anti-inflammatory cytokine IL-10 in colon tissue. Research showed that intestinal mucosal injury was increased in patients with IBD and colitis mice [35, 36]. Hence, preventing intestinal barrier dysfunction and promoting mucosal healing holds great promise for treating UC [37]. We found QYQC treatment effectively boosted the presence of goblet cells in intestinal mucosa. Moreover, QYQC treatment upregulated the expression of ZO-1, MUC2, and Claudin-4, which demonstrated its potential therapeutic effects on UC.

PPAR-γ, acts as the central coordinator of the intestinal barrier, has been reported to have decreased expression in intestinal tissues [11, 15]. Similarly, the PPAR-γ signaling pathway was found to be suppressed in colon tissue of DSS-induced colitis mice in our present study. However, QYQC intervention reactivated the PPAR-γ signaling pathway. In addition, PPAR-γ inhibited the overexpression of pro-inflammatory response genes by targeting transcription factors in the nucleus [38]. Interestingly, our study revealed that QYQC markedly increased nuclear levels of PPAR-γ rather than the cytoplasm. Further molecular docking results demonstrate that Baicalin, Paeoniflorin, Mollugin, and Imperatorin exhibit favorable binding with PPAR-γ, suggesting their potential as core components in QYQC for treating UC.

Patients with UC experience structural and functional impairments in intestinal mucosal mitochondria, affecting the TCA cycle pathway, and disrupting levels of energy metabolism-related products [39]. Furthermore, PPAR-γ enhances the mitochondrial TCA cycle, promotes energy supply to colonic epithelial cells while maintaining a low-oxygen environment in the intestine, and inhibits the growth of facultative anaerobes [12]. Our experimental results have demonstrated that QYQC could reactivate the expression of PPAR-γ signaling in colon tissue of colitis mice. In order to further verify whether QYQC-mediated upregulation of PPAR-γ signaling could improve colon energy metabolism in colitis mice, we then performed targeted energy metabolomics analysis in colon tissues of mice. The results indicated that QYQC reshaped the colonic metabolism, leading to significant changes in potential biomarkers like fructose, citric acid, and lactate levels in colonic tissues. The level of TCA cycle-related metabolites such as citric acid in the colonic tissue of UC patients was reduced, which could not meet the energy demands for the repair and maintenance of the intestinal mucosal barrier [40]. Our study found that QYQC upregulates the levels of TCA cycle-related metabolite citrate. Interestingly, the colonic tissue citric acid content is positively correlated with PPAR-γ but inversely correlated with the relative abundance of *Enterobacteriaceae*. More importantly, pathway analysis results revealed a remarkable improvement in energy metabolism pathways such as the TCA cycle, glyoxylate and dicarboxylate metabolism, as well as starch and sucrose metabolism in colitis mice after QYQC intervention. Therefore, activation of the PPAR-γ signaling pathway mediated by QYQC may enhance colonic epithelial cell mitochondrial energy metabolism represented by TCA cycle.

Mitochondria maintain a low-oxygen environment in the gut lumen through pathways such as the TCA cycle, OXPHOS, and fatty acid β-oxidation, thereby preserving the balance between gut luminal facultative anaerobes and obligate anaerobes [12]. We next thought to clarify whether QYQC-mediated improvement in mitochondrial energy metabolism can inhibit the proliferation of facultative anaerobes, we further analyzed the structure of the intestinal microbiota of mice in each group. Our current study observed that QYQC considerably suppressed the population of facultative anaerobes in DSS-treated mice, including *Erysipelatoclostridiaceae, Erysipelotrichaceae, Enterobacteriaceae, norank_o__RF39, Acholeplasmataceae, Streptococcaceae* and *Staphylococcaceae*. Furthermore, we also found that *Enterobacteriaceae* and *Erysipelatoclostridiaceae* had a significantly positive correlation with pro-inflammatory cytokines in colon tissue. These results suggested that the role of QYQC in alleviating DSS-induced colonic inflammation may partially result from its ability to reactivate the PPAR-γ signaling pathway, enhance mitochondrial energy metabolism, and subsequently inhibit the pathological growth of facultative anaerobes, particularly *Enterobacteriaceae.*

It is reported that increased *E. coli* exacerbated colitis in mice [41]. In line with this report, an elevated proportion of *E. coli* (phylum Proteobacteria, family *Enterobacteriaceae*) has been detected in the feces of DSS-treated mice. QYQC treatment reduced the proportion of *E. coli*, as well as a decrease in the concentration of LPS in colonic tissues. The overexpression of iNOS caused by PPAR-γ deficiency in intestinal epithelial cells leads to disturbance of the intestinal flora, manifested by an imbalance between facultative anaerobes and obligate anaerobics [42]. Consistent with previous findings, we observed a significant increase in iNOS expression in the DSS group, while QYQC intervention reduced the iNOS expression. These results suggested that QYQC intervention improved mitochondrial energy metabolism, inhibited the growth of facultative anaerobes (especially *E. coli*), and then ameliorated colon inflammation.

Finally, the PPAR-γ antagonist was used to confirm whether the effect of QYQC in improving colitis was mediated by PPAR-γ signaling. We found that the protective effects of QYQC on DSS-treated mice were abrogated by inhibition of PPAR-γ signaling by T0070907. Compared with QYQC group, the protein level of IL-1β in colonic tissue was significantly raised in QYQC+T group. The pathological lesions of the intestinal mucosa of QYQC+T group was also more severe than QYQC group. Furthermore, the protein expression of ZO-1 and MUC2 in colonic tissue was significantly reduced in QYQC+T group. Apart from the disrupted protective effect of intestine inflammation and the mucosal barrier, higher relative abundance of facultative anaerobes, along with increased expansion of *E. coli* and elevated levels of LPS, were observed when using PPAR-γ antagonist T0070907. These findings reiterated the potential mechanism of QYQC in improving UC by activating the PPAR-γ signaling pathway.

## Conclusions

**Fig. 10:**
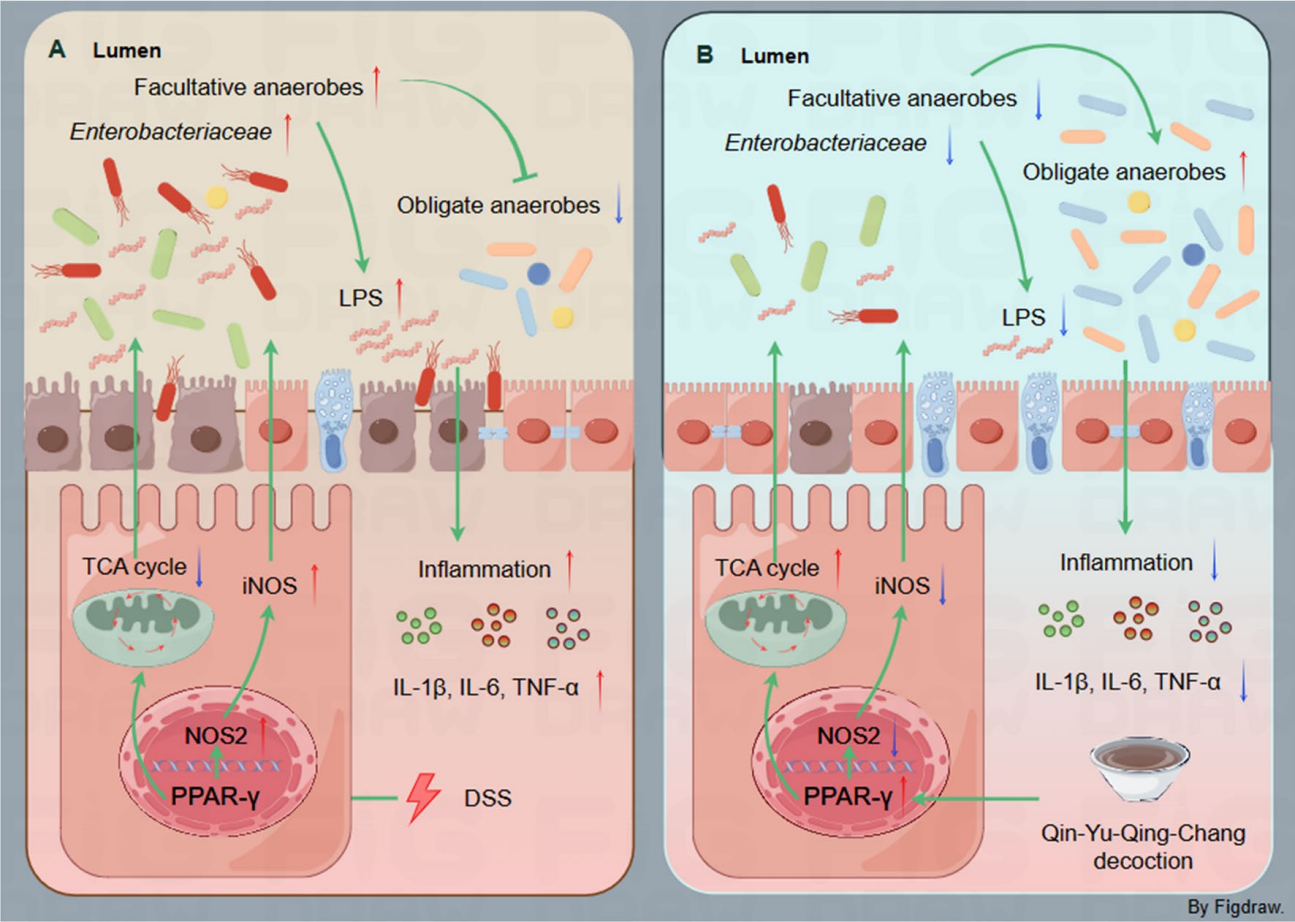
An overview of the mechanism diagram. QYQC ameliorates UC by activating PPAR-γ signaling and boosting the translocation of PPAR-γ into the nucleus, which enhances colonic epithelial cell mitochondrial energy metabolism and thereby curbing the uncontrolled proliferation of facultative anaerobes

In summary, our study unveiled that the favorable outcomes of QYQC in UC can be attributed to its regulation of the PPAR-γ signaling pathway. Notably, QYQC activated the PPAR-γ signaling pathway and increased the influx of PPAR-γ into the nucleus. This activation effectively heightened mitochondrial energy metabolism and prevented dysbiotic expansion of facultative anaerobes, including *E. coli* (Fig. 10).

## Supplementary Information

Additional file 1.

Additional file 2.

Additional file 3.

Additional file 4.

Additional file 5.

Additional file 6.

## Data Availability

All data in this study are available from the corresponding author upon reasonable request.

## Abbreviations

AB-PAS: Alcian blue/periodic acid-Schiff
BPC: Base peak chromatograms
CFU: Colony-forming units
DAI: Disease activity index
DSS: Dextran sulfate sodium
*E. coli*: *Escherichia coli*
ESI: Electrospray ionization
GSEA: Gene set enrichment analysis
H&E: Hematoxylin and eosin
IL: Interleukin
iNOS: Inducible nitric oxide synthase
KEGG: Kyoto encyclopedia of genes and genomes
LPS: Lipopolysaccharides
Muc2: Mucin2
MRM: Multiple reaction monitoring
OXPHOS: Oxidative phosphorylation
PCA: Principal component analysis
PCoA: Principal coordinates analysis
PPAR-γ: Peroxisome proliferator-activated receptor-γ
QCHS: Qing-Chang-Hua-Shi formula
QYQC: Qin-Yu-Qing-Chang decoction
RXR: Retinoid X receptor
TCA cycle: Citrate cycle
TNF-α: Tumor necrosis factor-α
UC: Ulcerative colitis
UHPLC: Ultra-high-performance liquid chromatography
ZO-1: Zonula occludens-1
5-ASA: 5-Aminosalicylic acid
