# Supplementary material for: Qin-Yu-Qing-Chang decoction reshapes colonic metabolism by activating PPAR-γ signaling to inhibit facultative anaerobes against DSS-induced colitis
Source: Chin Med. 2024 Sep 26;19:130. doi: 10.1186/s13020-024-01006-9 (PMC11425999; doi:10.1186/s13020-024-01006-9)

**Additional file 1**

*UHPLC-MS/MS analysis and pharmaceutical quality control of QYQC*

Sample analysis was conducted on the ultra-high-performance liquid chromatography (UHPLC) system (SHIMADZU-LC30) equipped with a Waters ACQUITY UPLC® HSS T3 column (2.1×100 mm, 1.8 µm, Milford, MA, USA). The mobile phase consisted of phase A: water (0.1% formic acid) and phase B: methanol. Gradient elution was used to investigate and screen the optimal separation effect. The elution conditions were as follows: 0-15 min, 10-100% B; 15-17 min, 100% B; 17-17.1 min, 100-10% B; 17.1-20 min, 10% B. The flow rate was 0.3 mL/min. The column temperature was 35°C, and the injection volume was 2 μL. The samples were separated using UHPLC.

Electrospray ionization was employed to scan the mass spectrometry in both positive and negative ion modes. The atomized gas was nitrogen and the flow rate was 800 L/h. The ion source voltage was 3000 V. The desolvation temperature was 500°C. The cone flow rate was 1 L/h and the ion source temperature was 150°C. The multiple reaction monitoring (MRM) scanning method was employed. The collision energy was 2500 V, and the carrier gas was argon. The dwell time was 0.02 s. The cone voltage and collision energy parameters were optimized according to MRM. The mass spectral acquisition time was set to 20 min, and the precursor scanning ranged from 100 to 1500 m/z. The conditions of MRM were listed in Table S1.

The concentration of each standard reagent was configured to a working concentration of 1 uM. The concentration of each marker compound in QYQC was determined by the ratio of the peak area of the QYQC marker component to the corresponding standard (the concentration of each marker compound in QYQC (uM)= the peak area of each marker compound in QYQC/ the peak area of each relevant standard reagent* the concentration of each relevant standard reagent (uM).

Table S1

The conditions of MRM.

| **NO.** | **Analytes** | **Parent ion (m/z)** | **Product ion (m/z)** | **Dwell (s)** | **Cone (V)** | **CE (V)** | **Rt (min)** |
| --- | --- | --- | --- | --- | --- | --- | --- |
| 1 | Baicalin | 447.20 | 271.20 | 0.043 | 30 | 30 | 6.51 |
| 2 | Anemoside B4 | 1222.10 | 455.40 | 0.043 | 20 | 40 | 6.61 |
| 3 | Astilbin | 451.30 | 147.10 | 0.043 | 20 | 15 | 5.69 |
| 4 | Paeoniflorin | 525.20 | 121.00 | 0.030 | -30 | -37 | 5.08 |
| 5 | Gallic Acid | 169.07 | 125.01 | 0.030 | -20 | -20 | 2.33 |
| 6 | Mollugin | 285.17 | 253.16 | 0.043 | 5 | 15 | 15.69 |
| 7 | Paeonol | 167.10 | 149.10 | 0.043 | 20 | 20 | 9.70 |
| 8 | Imperatorin | 271.20 | 203.10 | 0.043 | 20 | 15 | 12.01 |
| 9 | Glycyrrhizic acid | 823.90 | 453.40 | 0.043 | 30 | 35 | 8.58 |

Table S2

Information about the standard reagents.

| **Compound** | **Formula** | **Class** | **CAS No.** | **Cot No.** | **Purity** |
| --- | --- | --- | --- | --- | --- |
| Baicalin | C_21_H_18_O_11_ | Flavone glycoside | 21967-41-9 | B20570 | HPLC≥98% |
| Anemoside B4 | C_59_H_96_O_26_ | Triterpenoid saponin | 129741-57-7 | WKQ-0000071 | HPLC≥98% |
| Astilbin | C_21_H_22_O_11_ | Rhamnoside | 29838-67-3 | B20812 | HPLC≥98% |
| Paeoniflorin | C_23_H_28_O_11_ | Monoterpene glucoside | 23180-57-6 | B21148 | HPLC≥98% |
| Gallic Acid | C_7_H_6_O_5_ | Trihydroxybenzoic acid | 149-91-7 | B20851 | HPLC≥98% |
| Mollugin | C_17_H_16_O_4_ | Naphthopyran | 55481-88-4 | B20253 | HPLC≥98% |
| Paeonol | C_9_H_10_O_3_ | Subsituted phenol | 552-41-0 | B20266 | HPLC≥98% |
| Imperatorin | C_16_H_14_O_4_ | Furanocoumarin | 482-44-0 | WKQ-0000413 | HPLC≥98% |
| Glycyrrhizic acid | C_42_H_62_O_16_ | Triterpenoid saponin | 1405-86-3 | B20417 | HPLC≥98% |

Table S3

Detailed information on the concentration of the marker compounds.

| **Compound** | **RT (min)** | **Peak area (Standards)** | **Concentration (Standards, uM)** | **Peak area (QYQC)** | **Concentration (QYQC, μM)** |
| --- | --- | --- | --- | --- | --- |
| Baicalin | 6.51 | 97097480 | 1.00 | 119201376 | 1.227646 |
| Anemoside B4 | 6.61 | 112878 | 1.00 | 67130 | 0.594713 |
| Astilbin | 5.69 | 4954221 | 1.00 | 5787480 | 1.168192 |
| Paeoniflorin | 5.08 | 744658 | 1.00 | 199296 | 0.267634 |
| Gallic Acid | 2.31 | 650958 | 1.00 | 275869 | 0.423789 |
| Mollugin | 15.69 | 12577011 | 1.00 | 3064610 | 0.243668 |
| Paeonol | 9.70 | 12918076 | 1.00 | 4703908 | 0.364134 |
| Imperatorin | 12.01 | 134022608 | 1.00 | 23939926 | 0.178626 |
| Glycyrrhizic acid | 8.58 | 8704601 | 1.00 | 4770850 | 0.548084 |

Fig. S1. The extracted chromatograms of the marker compounds with chemical structure and PubChem CID under the multiple reaction monitoring (MRM) model in the standards (A) and QYQC (B).


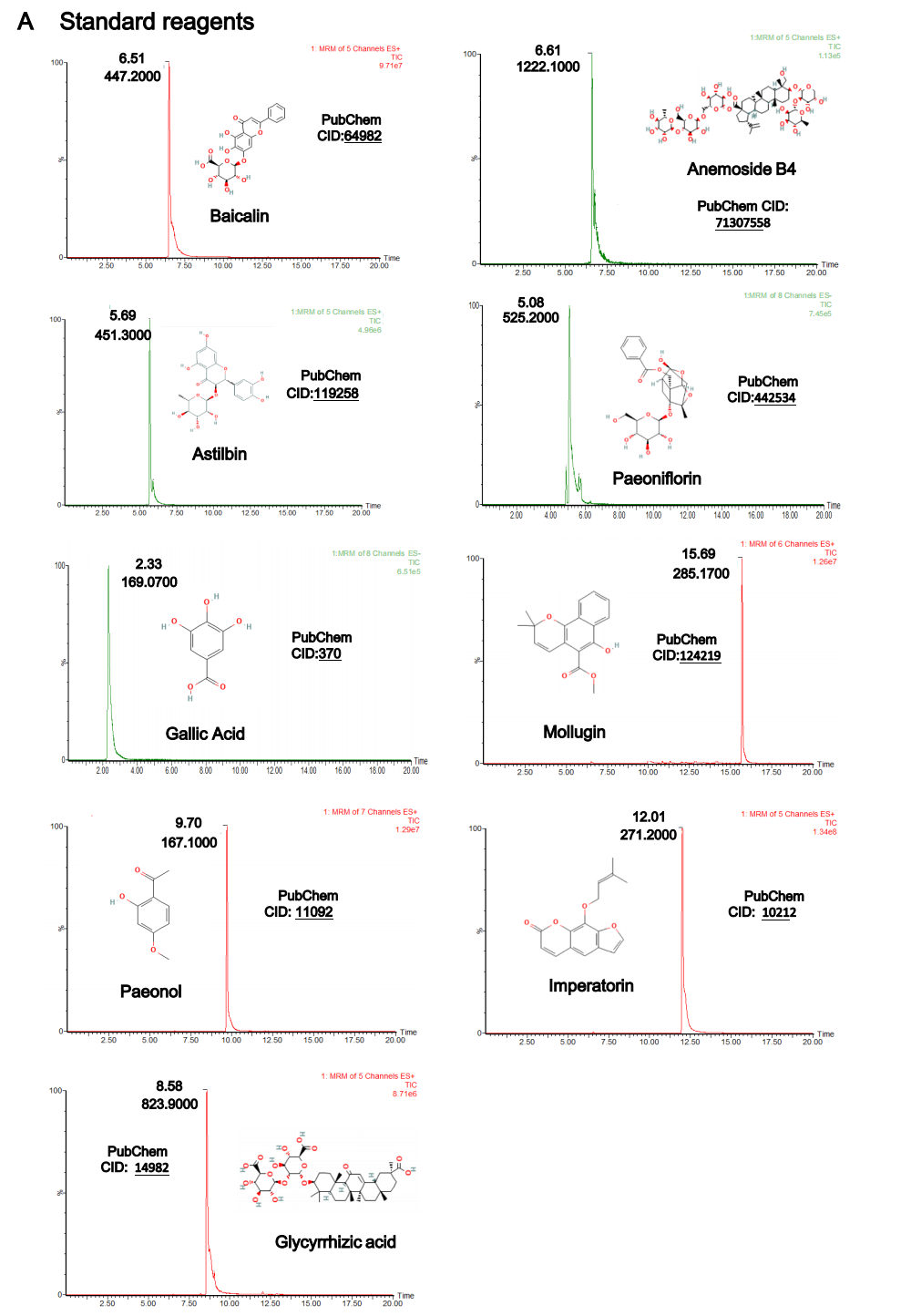


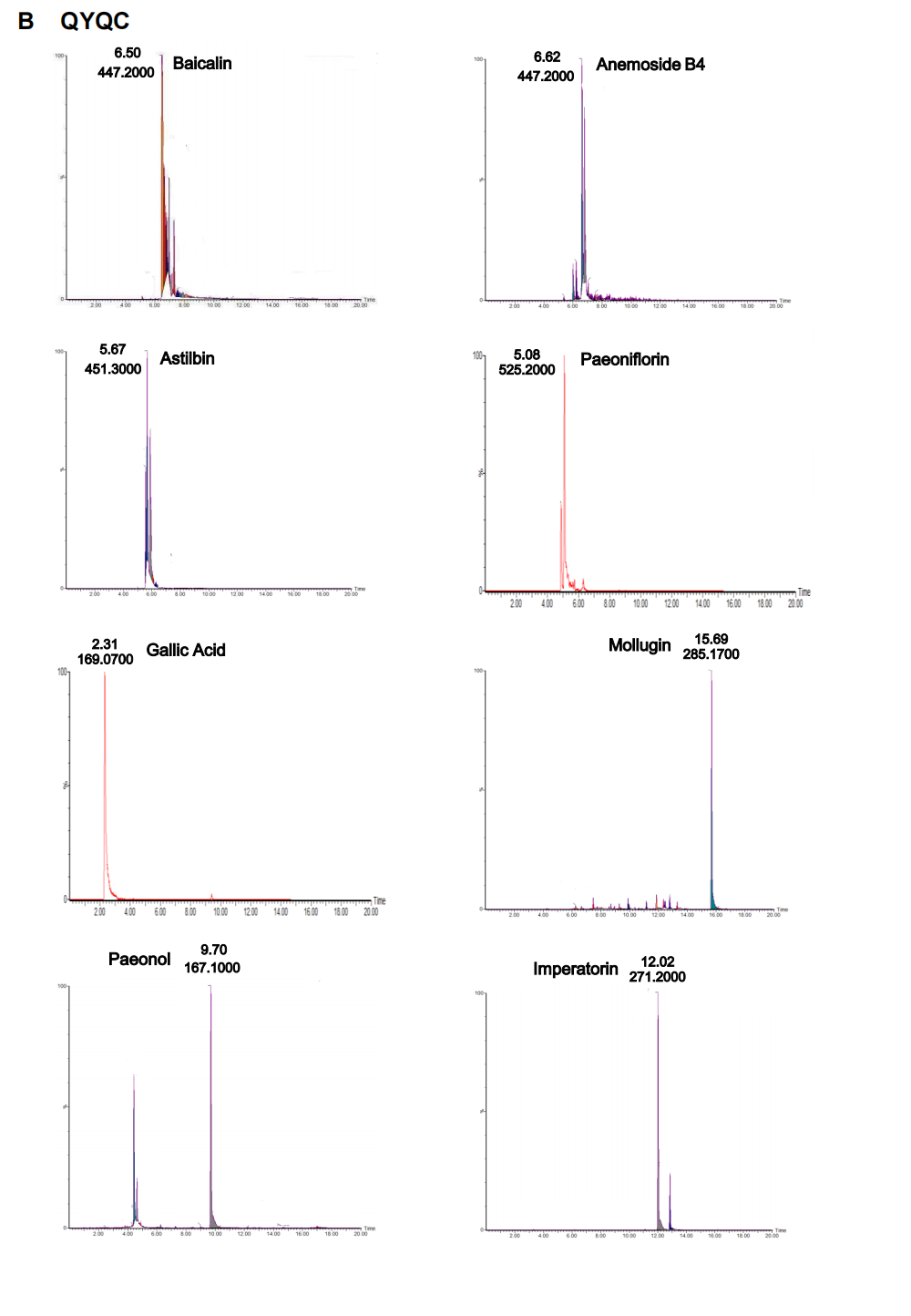


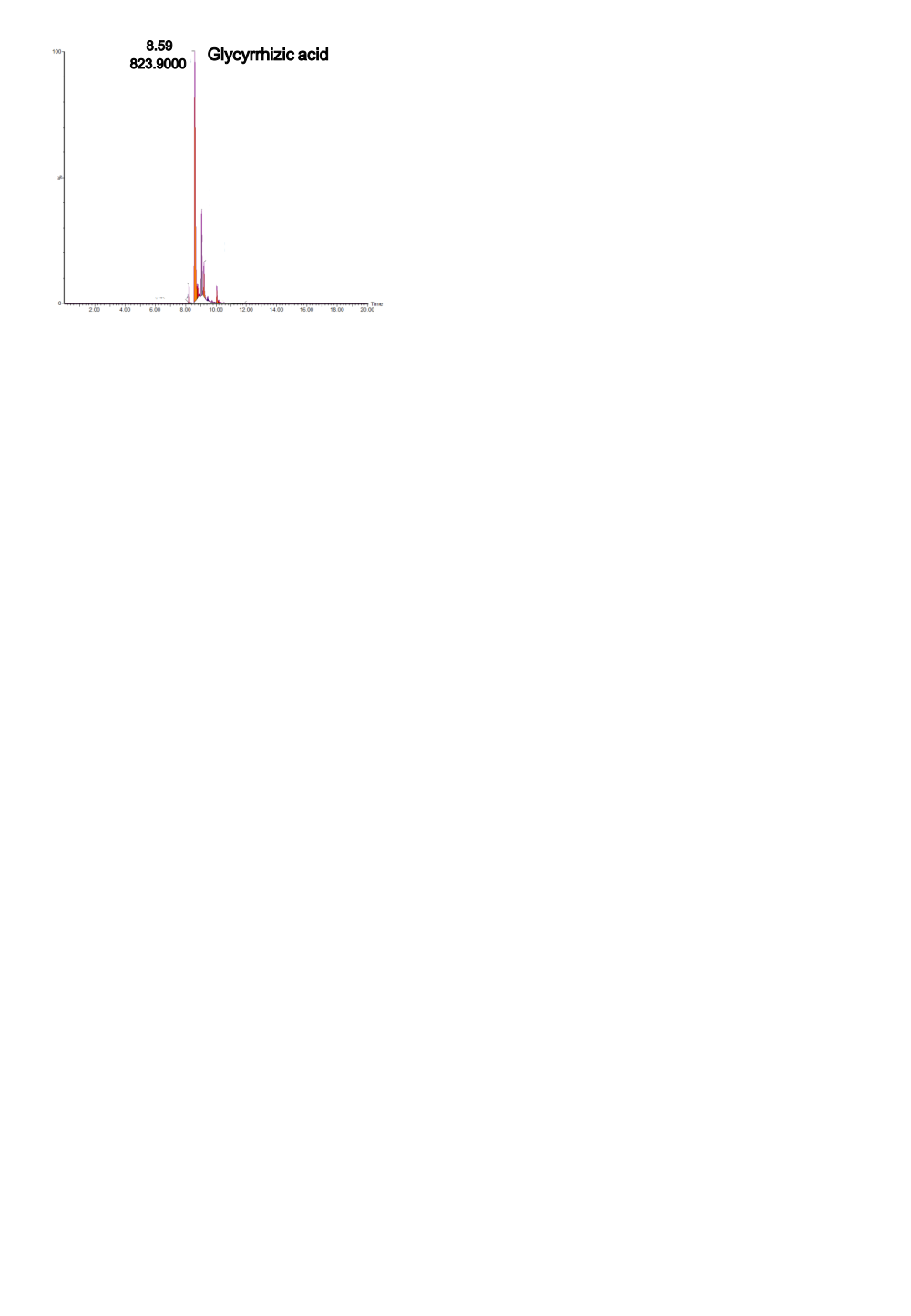

Supplement: Supplementary file 1 — Additional file 1. [file 13020_2024_1006_MOESM1_ESM.docx]
