# Supplementary material for: Qin-Yu-Qing-Chang decoction reshapes colonic metabolism by activating PPAR-γ signaling to inhibit facultative anaerobes against DSS-induced colitis
Source: Chin Med. 2024 Sep 26;19:130. doi: 10.1186/s13020-024-01006-9 (PMC11425999; doi:10.1186/s13020-024-01006-9)
Supplement: Supplementary file 2 — Additional file 2. [file 13020_2024_1006_MOESM2_ESM.docx]

**Additional file 2**

List of primers used in this study.

| **Genes** | **Primers** | **Primer Sequences (5′-3′)** |
| --- | --- | --- |
| **Primers for qPCR** |  |  |
| *Mouse-IL-1β* | F | 5’-AATGCCACCTTTTGACAGTGATG-3’ |
|  | R | 5’-GGAAGGTCCACGGGAAAGAC-3’ |
| *Mouse-TNF-α* | F | 5’-AAGGCCGGGGTGTCCTGGAG-3’ |
|  | R | 5’-AGGCCAGGTGGGGACAGCTC-3’ |
| *Mouse-IL-6* | F | 5’-CCACTTCACAAGTCGGAGGCTTA-3’ |
|  | R | 5’-AGTGCATCATCGTTGTTCATAC-3’ |
| *Mouse-IL-10* | F | 5’-CTTACTGACTGGCATGAGGATCA-3’ |
|  | R | 5’-GCAGCTCTAGGAGCATGTGG-3’ |
| *Mouse-ZO-1* | F | 5’-AGTTCTGCCCTCAGCTACCA-3’ |
|  | R | 5’-GCTTAAAGCTGGCAGTGTC-3’ |
| *Mouse-MUC-2* | F | 5’-TGTGTTTCAGGCTCCATCAC-3’ |
|  | R | 5’-TGCAGCCATTGTAGGAAATC-3’ |
| *Mouse-Claudin4* | F | 5’-GGGGATCATCCTGAGTTGTG-3’ |
|  | R | 5’-CACTGCATCTGACCTGTGCT-3’ |
| *Mouse-Cpt1b* | F | 5’-GAGTTTGTCCTGGGCACTGA-3’ |
|  | R | 5’-ACGAGTTCTCGATGGCTTCC-3’ |
| *Mouse-Fabp3* | F | 5’-GTGACAGCAGATGACCGGAA-3’ |
|  | R | 5’-TTAGTGTTGTCTCCTGCCCG-3’ |
| *Mouse-IIK* | F | 5’-ATGCACTCAATAGCCGCAGT-3’ |
|  | R | 5’-TGTCTGCTGAGCGTCTGTTT-3’ |
| *Mouse-Slc27a2* | F | 5’-TGAATGTGTATGGCGTGCCT-3’ |
|  | R | 5’-AGGTACTCCGCGATGTGTTG-3’ |
| *Mouse-Pck1* | F | 5’-TGAAAGGCCGCACCATGTAT-3’ |
|  | R | 5’-GCACAGATATGCCCATCCGA-3’ |
| *Mouse-Adipoq* | F | 5’-AGCCGCTTATGTGTATCGCT-3’ |
|  | R | 5’-GAGTCCCGGAATGTTGCAGT-3’ |
| *Mouse-Slc27a1* | F | 5’-GGCCACCATTCCTACAGCAT-3’ |
|  | R | 5’-ACACAGTCATCCCAGAAGCG-3’ |
| *Mouse-PPAR-γ* | F | 5’-TACTGCCGTTTTCACAAGTGC-3’ |
|  | R | 5’-AGGTCGTGTTCACAGGTAAGA-3’ |
| *Mouse-iNOS* | F | 5’-GCCCCTGGAAGTTTCTCTTC-3’ |
|  | R | 5’-CTGAGAACAGCACAAGGGGT-3’ |
| *Mouse-β-actin* | F | 5’-CTCATGAAGATCCTGACCGAG-3’ |
|  | R | 5’-AGTCTAGAGCAACATAGCACAG-3’ |
