# Supplementary material for: Qin-Yu-Qing-Chang decoction reshapes colonic metabolism by activating PPAR-γ signaling to inhibit facultative anaerobes against DSS-induced colitis
Source: Chin Med. 2024 Sep 26;19:130. doi: 10.1186/s13020-024-01006-9 (PMC11425999; doi:10.1186/s13020-024-01006-9)
Supplement: Supplementary file 3 — Additional file 3. [file 13020_2024_1006_MOESM3_ESM.docx]

**Additional file 3**

*RNA sequencing*

RNA quantity, purity, cDNA Synthesis, and Library Preparation were conducted following the manufacturer's instructions. The cDNA library had an average insert size of 300±50 base pairs for the final product. At last, we conducted paired-end sequencing (PE150) using an Illumina Novaseq™ 6000 under the vendor's prescribed protocol.

Quality assessment of raw data was performed using Fastp software. HISAT2 (v2.1.0) was employed for mapping reads to the reference genome of [Mus musculus](https://www.ncbi.nlm.nih.gov/nuccore/?term=IL-6) mm10. The mapped reads from each sample were assembled using StringTie with default parameters. Transcriptomes from all samples were then merged to reconstruct a comprehensive transcriptome using gffcompare. StringTie was used to compute expression levels of all transcripts, measured as FPKM. Differentially expressed mRNAs between samples were selected based on specific criteria using the R package edgeR (fold change > 1.2, *p* < 0.05).
