# Supplementary material for: Qin-Yu-Qing-Chang decoction reshapes colonic metabolism by activating PPAR-γ signaling to inhibit facultative anaerobes against DSS-induced colitis
Source: Chin Med. 2024 Sep 26;19:130. doi: 10.1186/s13020-024-01006-9 (PMC11425999; doi:10.1186/s13020-024-01006-9)
Supplement: Supplementary file 4 — Additional file 4. [file 13020_2024_1006_MOESM4_ESM.docx]

**Additional file 4**

*Gut microbiota analysis*

Total DNA from ileocecum feces was extracted and the concentration and purity of bacterial DNA were detected. The V3-4 region of the bacterial 16S rRNA genes were amplified with specific primers. All PCR reactions were performed on ABI GeneAmp® 9700 PCR thermocycler system (ABI, Foster City, CA, USA) and quantified using Quantus™ Fluorometer (Promega, Madison, WI, USA).

Equimolarly pooled purified amplicons were subjected to paired-end sequencing on an Illumina MiSeq PE300 platform (Illumina, San Diego, USA), following standard protocols conducted by Majorbio Bio-Pharm Technology Co. Ltd. (Shanghai, China).
