# Supplementary material for: Qin-Yu-Qing-Chang decoction reshapes colonic metabolism by activating PPAR-γ signaling to inhibit facultative anaerobes against DSS-induced colitis
Source: Chin Med. 2024 Sep 26;19:130. doi: 10.1186/s13020-024-01006-9 (PMC11425999; doi:10.1186/s13020-024-01006-9)
Supplement: Supplementary file 5 — Additional file 5. [file 13020_2024_1006_MOESM5_ESM.docx]

**Additional file 5**

*Energy metabolomic analysis*

The instrument conditions and parameter settings for UPLC-MS/MS analysis were as follows: The Acquity-I Xevo TQ-S liquid chromatography-tandem mass spectrometer (Waters Corp., Milford, MA, USA), equipped with a BEH C18 1.7 µm analytical column (2.1 × 100 mm), was used for the detection of energy metabolism-related metabolites. The column temperature was 40°C, and the sample manager temperature was 10°C. The mobile phase consists of A: 5mM DIPEA aqueous solution, and B: ACN: IPA=7:3. The elution conditions were as follows: 0-1 min (1% B), 1-9.5 min (1-15% B), 9.5-13 min (15-62% B), 13-14 min (62-100%B), 14-16 min (100% B), 16-16.2min (100-1%B), 16.2-18min (1%B). The flow rate was 0.3 mL/min and the injection volume was 5.0 µL. The capillary voltage of the mass spectrometer was 3kV (ESI-). The source temperature was set to 150 °C, the desolvation temperature was 500 °C, and the desolvation gas flow was set at 1000 L/Hr.

Reagent blanks and mixed quality control samples were added to monitor potential contamination and ensure data quality during the analysis process. Processing of the raw data files from UPLC-MS/MS, which included tasks such as peak identification, integration, calibration, and quantification for each metabolite, was performed using MassLynx software (V4.1, Waters, Milford, MA, USA). Subsequent statistical analysis was conducted using iMAP software (V1.0, Metabo-Profile, Shanghai, China).
