# Supplementary material for: Qin-Yu-Qing-Chang decoction reshapes colonic metabolism by activating PPAR-γ signaling to inhibit facultative anaerobes against DSS-induced colitis
Source: Chin Med. 2024 Sep 26;19:130. doi: 10.1186/s13020-024-01006-9 (PMC11425999; doi:10.1186/s13020-024-01006-9)
Supplement: Supplementary file 6 — Additional file 6. [file 13020_2024_1006_MOESM6_ESM.docx]

**Additional file 6**


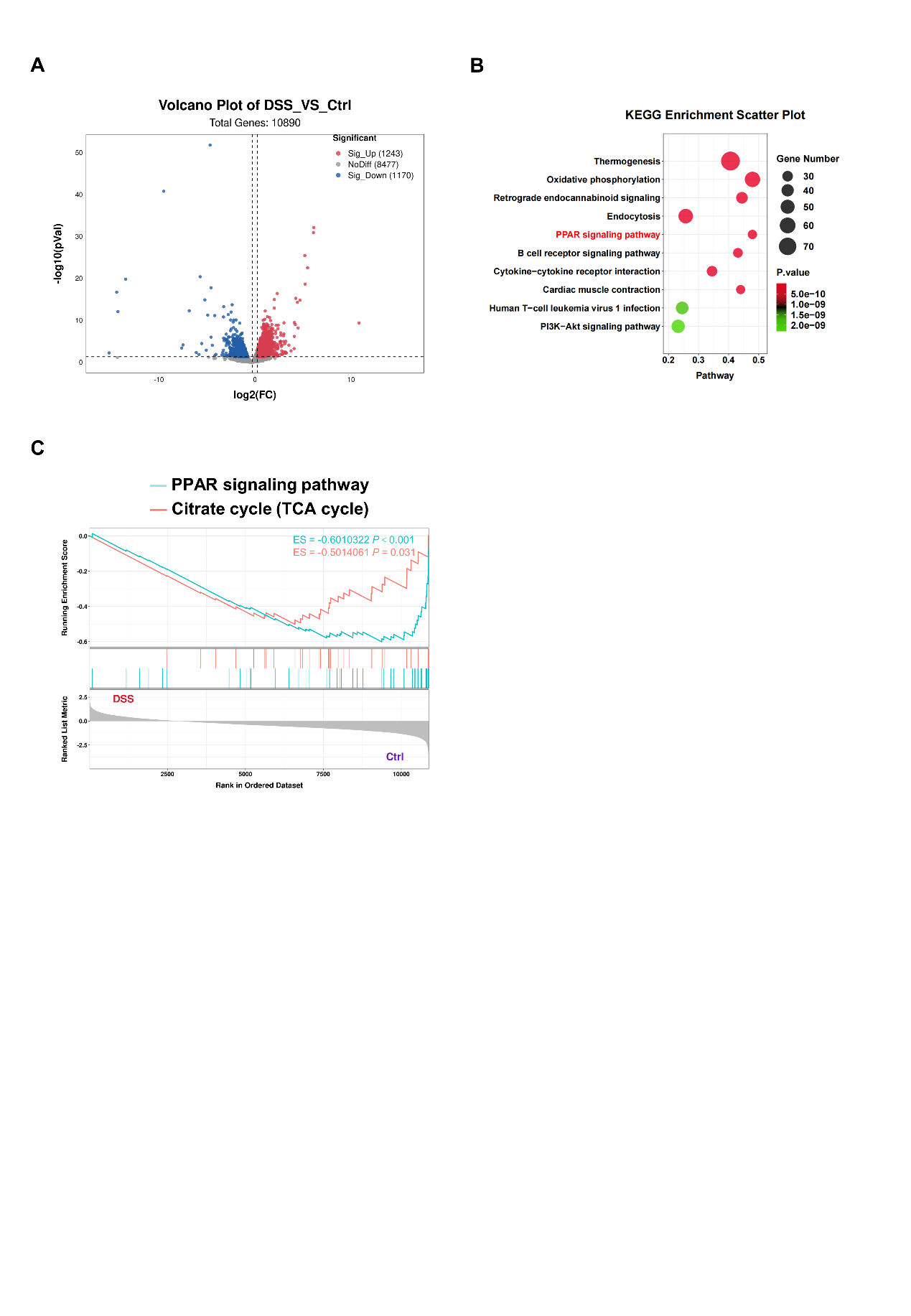


Fig. S2. The PPAR signaling pathway in the colon tissue of DSS group is inhibited. (A) Volcano plot of differentially expressed genes between DSS and Ctrl group (n=3). (B) KEGG pathway enrichment analysis of the most significantly changed pathways among mice from the DSS and Ctrl group (n=3). (C) GSEA of PPAR signaling pathway and TCA cycle between DSS and Ctrl groups (n=3).


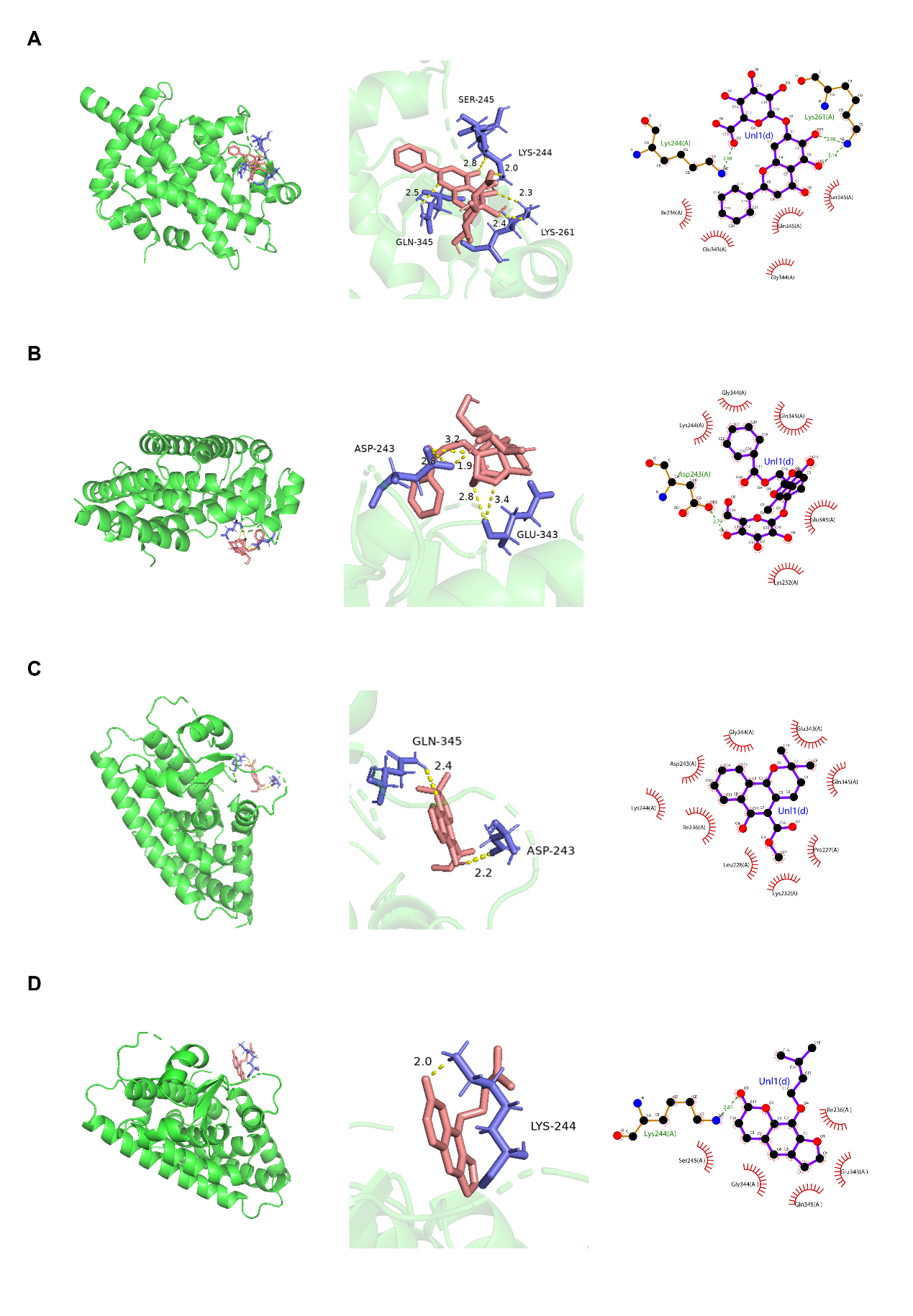


Fig. S3. The binding sites of representative compounds of QYQC with PPAR-γ identified through molecular docking. (A) Baicalin-PPAR-γ. (B) Paeoniflorin-PPAR-γ. (C) Mollugin-PPAR-γ. (D) Imperatorin-PPAR-γ.


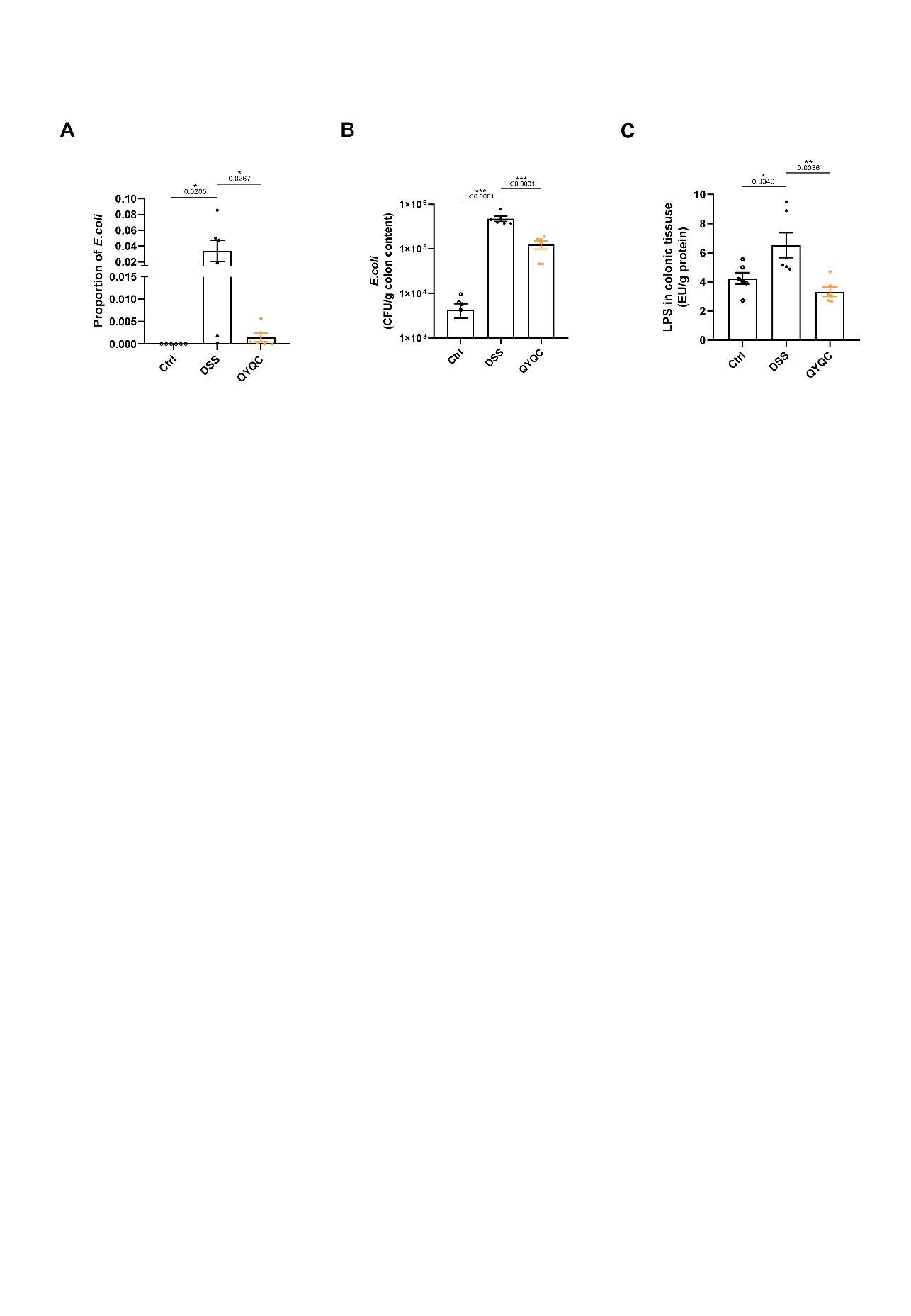


Fig. S4. QYQC inhibits the expansion of *Enterobacteriaceae*. (A) Relative *E. coli* abundance at the specie level (n = 6). (B) Bacterial load in colon content (n = 6). (C) Colonic tissue LPS concentration (n = 6). Data are expressed as Mean ± SEM. **p* < 0.05, ***p* < 0.01, ****p* < 0.001.


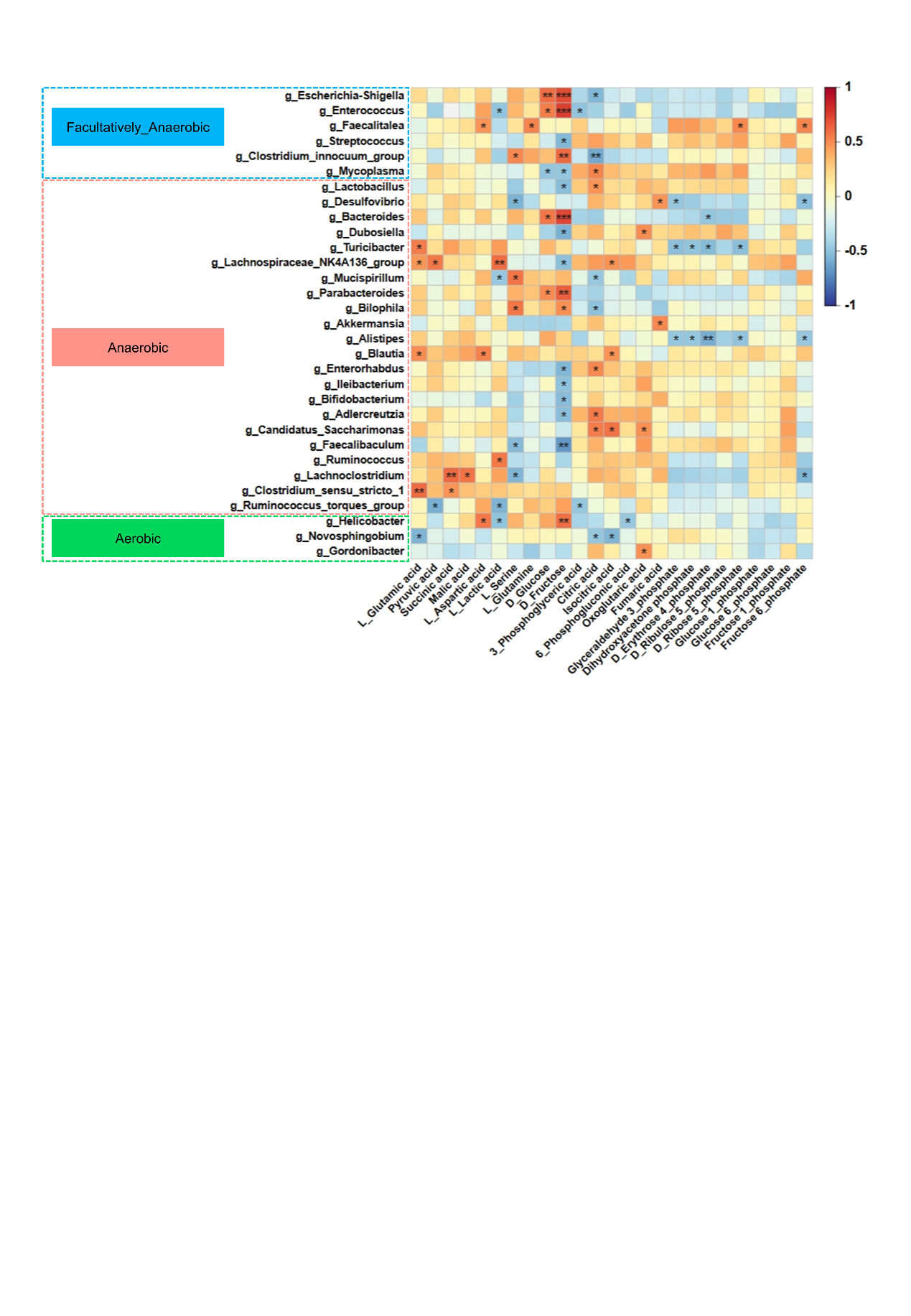


Fig. S5. The relationship between gut microbiota and colon metabolites. **p* < 0.05, ***p* < 0.01, ****p* < 0.001.


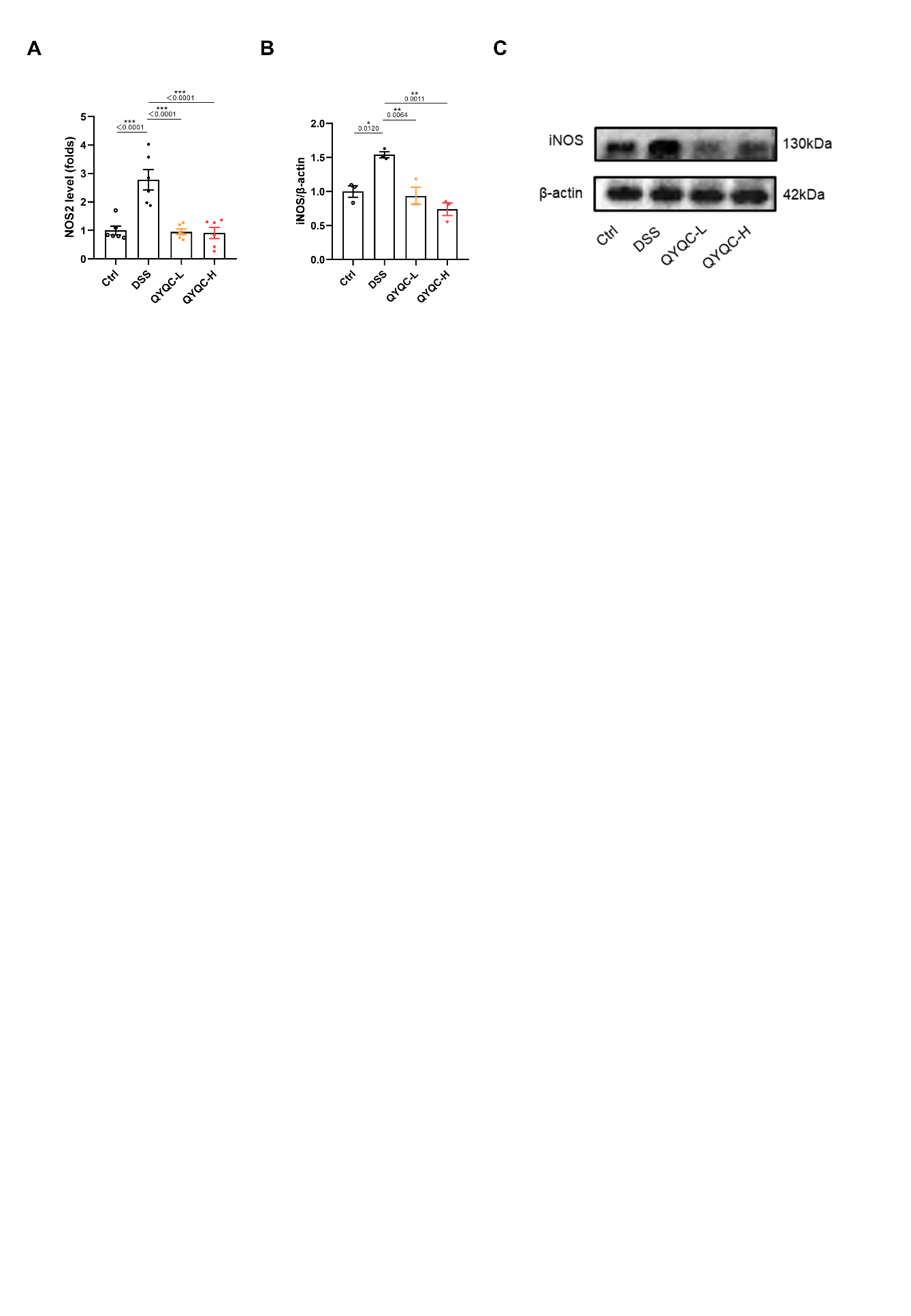


Fig. S6. QYQC inhibits the expression of iNOS. (A) Relative mRNA expression of iNOS. (B-C) Relative protein expression of iNOS. Data are expressed as Mean ± SEM. **p* < 0.05, ***p* < 0.01, ****p* < 0.001.


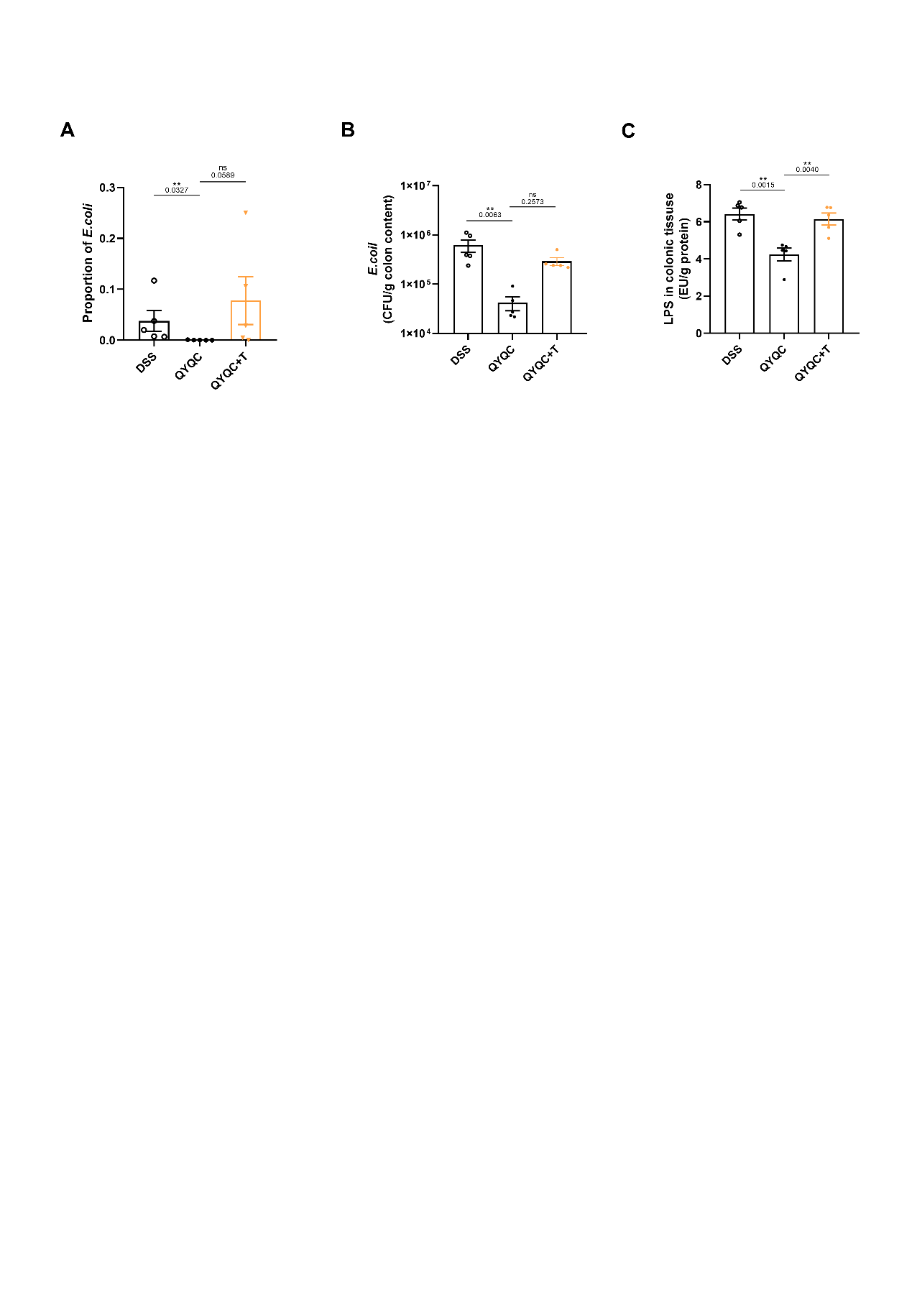


Fig. S7. The effect of QYQC on *Enterobacteriaceae* is reversed by PPAR-γ antagonist. (A) Relative *E. coli* abundance at the species level (n = 5). (B) Bacterial load in colon content (n = 5). (C) Colonic tissue LPS concentration (n = 5). Data are expressed as Mean ± SEM. ***p* < 0.01.

Table S4

Results of the target and core compounds for molecular docking.

| **Target** | | **PDB ID** | **Compound** | | | **Affinity (kcal/mol)** |
| --- | --- | --- | --- | --- | --- | --- |
| PPAR-γ | | 6KEY | Baicalin | | | -5.51 |
|  | |  | Paeoniflorin | | | -5.29 |
|  | |  | Mollugin | | | -4.86 |
|  | |  | Imperatorin | | | -4.84 |
|  |  | | |  |  | |
